# Effects of cannabidiol, with and without ∆9-tetrahydrocannabinol, on anxiety-like behavior following alcohol withdrawal in mice

**DOI:** 10.3389/fnins.2024.1375440

**Published:** 2024-06-18

**Authors:** Mariam Melkumyan, Vibha M. Annaswamy, Alexandra M. Evans, Opeyemi F. Showemimo, Zari E. McCullers, Dongxiao Sun, Terrence E. Murphy, Kent E. Vrana, Amy C. Arnold, Wesley M. Raup-Konsavage, Yuval Silberman

**Affiliations:** ^1^The Pennsylvania State University College of Medicine, Department of Neural and Behavioral Sciences, Hershey, PA, United States; ^2^The Pennsylvania State University College of Medicine, Department of Pharmacology, Hershey, PA, United States; ^3^The Pennsylvania State University College of Medicine, Department of Public Health Sciences, Hershey, PA, United States

**Keywords:** alcohol withdrawal, anxiety, Cannabidiol (CBD), ∆9-tetrahydrocannabinol (THC), open field test, microglia, astrocytes

## Abstract

**Introduction:**

Alcohol use disorder (AUD) is commonly associated with anxiety disorders and enhanced stress-sensitivity; symptoms that can worsen during withdrawal to perpetuate continued alcohol use. Alcohol increases neuroimmune activity in the brain. Our recent evidence indicates that alcohol directly modulates neuroimmune function in the central amygdala (CeA), a key brain region regulating anxiety and alcohol intake, to alter neurotransmitter signaling. We hypothesized that cannabinoids, such as cannabidiol (CBD) and ∆9-tetrahydrocannabinol (THC), which are thought to reduce neuroinflammation and anxiety, may have potential utility to alleviate alcohol withdrawal-induced stress-sensitivity and anxiety-like behaviors via modulation of CeA neuroimmune function.

**Methods:**

We tested the effects of CBD and CBD:THC (3:1 ratio) on anxiety-like behaviors and neuroimmune function in the CeA of mice undergoing acute (4-h) and short-term (24-h) withdrawal from chronic intermittent alcohol vapor exposure (CIE). We further examined the impact of CBD and CBD:THC on alcohol withdrawal behaviors in the presence of an additional stressor.

**Results:**

We found that CBD and 3:1 CBD:THC increased anxiety-like behaviors at 4-h withdrawal. At 24-h withdrawal, CBD alone reduced anxiety-like behaviors while CBD:THC had mixed effects, showing increased center time indicating reduced anxiety-like behaviors, but increased immobility time that may indicate increased anxiety-like behaviors. These mixed effects may be due to altered metabolism of CBD and THC during alcohol withdrawal. Immunohistochemical analysis showed decreased S100β and Iba1 cell counts in the CeA at 4-h withdrawal, but not at 24-h withdrawal, with CBD and CBD:THC reversing alcohol withdrawal effects..

**Discussion:**

These results suggest that the use of cannabinoids during alcohol withdrawal may lead to exacerbated anxiety depending on timing of use, which may be related to neuroimmune cell function in the CeA.

## Introduction

Anxiety disorders are highly prevalent in the United States, affecting around 15% of individuals annually (Terlizzi, 2020). Anxiety disorders are highly comorbid with alcohol use disorders (AUD), and individuals with comorbid anxiety and AUD have poor treatment outcomes and high alcohol use relapse rates (Schellekens et al., 2015). Evidence indicates that chronic alcohol use and withdrawal increases anxiety and stress-sensitivity, which increases alcohol use via a negative reinforcement cycle for perpetuation of AUD risk (Kushner et al., 2000, 2005; Anker and Kushner, 2019; Castelli et al., 2022). Further, external stressors, particularly during withdrawal, may lead individuals suffering from AUD to relapse to alleviate stress (Koob et al., 2020). Therefore, it is important to find treatment options that will help decrease anxiety and stress-sensitivity in the context of alcohol withdrawal, which may ultimately reduce further alcohol use and relapse.

Cannabinoids have potential as therapeutics for anxiety- and stress-related disorders (Lookfong et al., 2023). The most widely used phytocannabinoids are cannabidiol (CBD) and ∆^9^-tetrahydrocannabinol (THC; Tambaro and Bortolato, 2012). THC is psychotropic, producing emotional and cognitive changes, appetite stimulation, and analgesia. At low doses, THC has been shown to decrease stress reactivity and amygdala activation when viewing fearful faces (Phan et al., 2008; Childs et al., 2017; Raymundi et al., 2020). However, at a higher dose, THC may increase anxiety, negative affective states, and reactivity of the amygdala (Bhattacharyya et al., 2010, 2017; Childs et al., 2017; Raymundi et al., 2020). CBD, on the other hand, is not psychotropic but can be anxiolytic (García-Gutiérrez et al., 2020) and can both reduce fear-related behaviors and produce antidepressant and antipsychotic effects (Elsaid and Le Foll, 2020; Gasparyan et al., 2021). These effects may also be sex-specific depending on the length of treatment time (acute vs. chronic; Kasten et al., 2019). CBD has also been shown to alter the potency and efficacy of THC, and potentially reduce THC’s psychoactive effects, although findings are mixed (Laprairie et al., 2015; Freeman et al., 2019). Therefore, we sought to test whether CBD might reduce anxiety-like behaviors differently in the presence of low dose THC than without THC in mouse models.

We examined alcohol-withdrawal induced anxiety-like behaviors in this study, as the role of CBD and THC on these behaviors is less studied. In terms of alcohol use, CBD has been shown to reduce alcohol consumption and seeking in a dose-dependent manner in mouse models (Viudez-Martínez et al., 2018). Additionally, studies have shown that alcohol withdrawal induces changes in endocannabinoid mRNA expression in a sex-dependent manner in various brain regions, including the basolateral amygdala and the ventromedial prefrontal cortex (Henricks et al., 2017). A recent study explored the effect of varying doses of CBD on alcohol withdrawal symptoms and saw a reduction in anxiety-like symptoms at a high dose (40 mg/kg; Gasparyan et al., 2023). It is worth noting that most people who use cannabis often use mixtures of CBD and THC, even if the product they are using is advertised as having only CBD or only THC (Chandra et al., 2017). CBD:THC ratios affect alcohol consumption, with low CBD:high THC ratio (above 1:10 CBD:THC ratio) users reporting the most alcohol drinking, while medium (between 1:1 and 1:10 CBD:THC) and high CBD:low THC (higher than 1:1 CBD:THC) ratio users report the least alcohol consumption (Karoly et al., 2020). Given the impact of CBD on THC efficacy and potency in relation to anxiety-like behaviors and the interaction of CBD:THC dosing on alcohol intake in humans, here we sought to examine the impact of high CBD:low THC ratio on alcohol-withdrawal induce anxiety-like behaviors.

CBD and THC might alter alcohol withdrawal-induced behaviors through a variety of mechanisms. Of interest, it is known that alcohol exposure and stress both induce and are affected by increased neuroinflammatory activity in the brain (Won and Kim, 2020; Zheng et al., 2021; Anand et al., 2022; León-Rodríguez et al., 2022). Studies have also shown that cannabinoids can mitigate neuroinflammation produced by alcohol exposure and reduce alcohol drinking (Zhou et al., 2016; García-Baos et al., 2021). In addition to neuroinflammatory effects, the cannabinoid system is linked to alcohol-induced alterations to glutamatergic and GABAergic neurotransmission in the central amygdala (CeA), a region heavily involved in regulating alcohol intake and anxiety-like behaviors (Roberto et al., 2010; Ramikie et al., 2014; Kirson et al., 2018; Melkumyan and Silberman, 2022). Studies from our laboratory have shown that the function of astrocytes is critical in alcohol-induced increases in glutamatergic neurotransmission in the CeA (Melkumyan et al., 2022). The cannabinoid system is thought to significantly impact neuroimmune activity, with CB1 and CB2 receptors both being highly expressed on microglia and astrocytes (Crowe et al., 2014). These findings suggest that the cannabinoid system and alcohol may be acting in concert to alter neurotransmission in the CeA, leading to changes in anxiety-like behavior.

Therefore, in these studies, we hypothesized that (1) mice will have increased baseline anxiety at two withdrawal timepoints (4-and 24-h withdrawal from chronic intermittent ethanol (CIE) vapor exposure) and this anxiety will be exacerbated in the presence of an additional stressor, and (2) the CBD alone and that of a mixture of high CBD and low THC (3:1 CBD:THC ratio) will reduce baseline anxiety-like behaviors in mice during 4-h and 24-h withdrawal in the presence or absence of additional stressors. In terms of neuroimmune cell activity we hypothesized that (1) alcohol withdrawal will lead to altered Iba1 and S100β cell counts in the CeA, and (2) the cannabinoids would rescue Iba1 and S100β changes in the CeA after a 4-and 24-h withdrawal from alcohol as a potential mechanism for reduced anxiety-like behaviors. To test these hypotheses, mice went through a 2-week CIE vapor inhalation protocol and were tested for anxiety-like behavior in the open field test, with and without pretest restraint stress exposure. We found increased anxiety at 4-h withdrawal, with CBD and 3:1 CBD:THC treatment further exacerbating anxiety-like behaviors while not having significant impact on stress-exposed mice at this time point. At 24-h, there was no effect of CBD alone while 3:1 CBD:THC reduced anxiety-like behaviors, which may be related to altered CBD and THC metabolism. Further, we found disrupted Iba1 morphology and density as well as decreased S100β cell counts at 4-h withdrawal, with no effect in 24-h withdrawal, and divergent impact of CBD and THC on Iba1 and S100β immunohistochemical markers at ths1ese time points.

## Methods

### Animals

248 adult (7–12 weeks old) male and female C57Bl/6 J mice (Jackson Laboratory) were used throughout the experiments. Mice were housed in groups of three to five and given *ad libitum* rodent chow (Teklad Global 18% Protein Rodent Diet, 2018S) and water in a 12/12 h light/dark cycle (lights on at 7 am). For the air control group (reference level for alcohol withdrawal), a total of 86 mice (50 male, 36 female) were used. For the alcohol vapor exposure groups, a total of 143 mice were used: 83 mice (53 male, 30 female) in the 24-h withdrawal group, and 60 mice (30 male, 30 female) in the 4-h withdrawal group. Of the 248 mice, 229 mice underwent the behavioral paradigm. A total of 19 mice (10 males and 9 females) did not undergo the behavioral paradigm and were used for plasma analysis of CBD and THC content 45 min post-injection. An additional 37 mice were used for plasma analysis of CBD and THC content at 80- and 140-min post-injection (see “Plasma collection and analysis” section below).

### Chronic intermittent ethanol exposure

CIE vapor inhalation is a widely accepted method to induce alcohol dependence and for the study of alcohol-withdrawal induced anxiety-like behaviors (Becker and Lopez, 2004). CIE exposures were performed in an ethanol vapor chamber system (La Jolla Alcohol Research, Inc.) to ensure equal levels of alcohol exposure per mouse. Mice were interperitoneally (i.p.) injected with a mixture of 1 mmol/kg pyrazole (Acros, 131,740,250) and 1 mg/kg alcohol (Pharmco, 111,000,190) immediately prior to being placed in the vapor chamber to reduce alcohol metabolism and maintain the desired blood ethanol concentration (130-200 mg/dL). Mice were exposed to vapors for 16 h/day, 4 days/week, for 2 weeks (on Monday, Tuesday, Thursday, and Friday). 95% ethanol was volatilized at 20 mg/L with airflow through the chambers maintained at 25 L/min with volatilization at 1.5 L/min. Mice had *ad libitum* access to rodent chow and water throughout the CIE exposures. Air control mice received i.p. injections of 1 mmol/kg pyrazole but were not exposed to alcohol vapor or injections. The behavioral paradigm started 4- or 24-h after the end of the last vapor exposure. The start time of the behavioral testing was consistent (~9-10 am) across all the groups (air control, 4-h withdrawal, 24-h withdrawal). To keep the behavioral testing at a consistent time, the time of the start of vapor exposure varied between the 4-h (~2 pm start) and 24-h (~5 pm start) withdrawal groups.

### Cannabinoid treatment and behavioral assessment of anxiety-like behaviors

**Figure 1:**
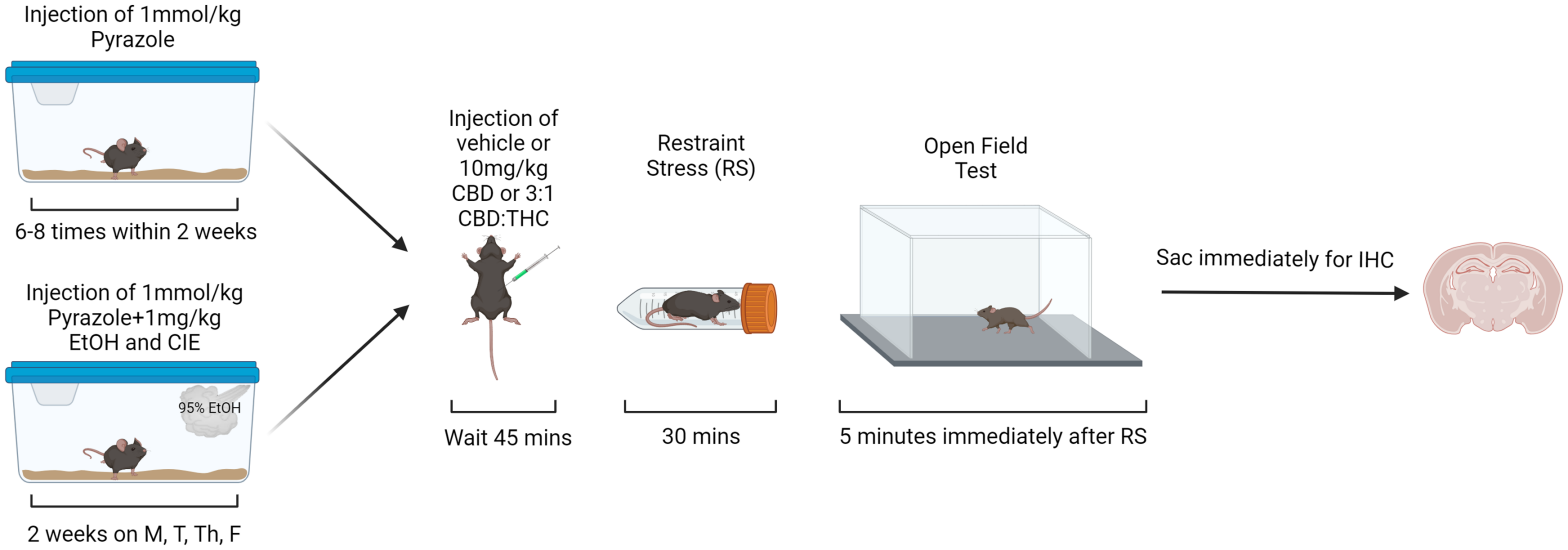
Behavioral paradigm for air control and alcohol exposed mice. Created with BioRender.

For the behavioral paradigm in the open field test, the mice were divided into six groups (see Table 1). Thirty mins prior to the start of behavioral assessments (occurring between 9 and 10 am), mice were brought to the testing room, weighed, and singly housed with no food or water access. The cages were covered with a tarp and the mice were left to habituate to the testing environment for 30 min prior to the start of the behavioral assessments. For behavioral assessments, the mice were i.p. injected with either vehicle (1:1:18 DMSO:Tween:Saline), 10 mg/kg CBD, or 10 mg/kg 3:1 CBD:THC (Cayman Chemical, Ann Arbor, MI) mixture (7.5 mg/kg CBD, 2.5 mg/kg THC). Forty-five mins after the injections, stressed mice were placed in a restraint tube for 30 min, while unstressed mice were kept in their cage for 30 min. During these 30 min, all cages were uncovered regardless of stress condition. The additional factor of restraint stress was used to mimic the translational aspect of alcohol dependence, where individuals in withdrawal may be more sensitive to external stressors. Such enhanced stress-responsiveness is thought to be a critical factor in continued alcohol use. Therefore, if cannabinoids can reduce reactivity to external stressors, they may be a therapeutic option in reducing alcohol use. Immediately after the stressor, mice were put in the open field arena (lux ~450) for 5 min (Figure 1). For the open field test (OFT), the time in center, total time immobile, distance traveled, and ratio of time spent immobile in the center were measured. Videos of the OFT were taken using ANY-maze USB camera connected to ANY-Maze 7.1, which was used to analyze the mouse behavior. The automated analysis was checked for accuracy by an experimenter blinded to the condition.

**Table 1:** Groups in the behavioral paradigm.

	Air		4-h withdrawal		24-h withdrawal	
	No stress	Stress	No stress	Stress	No stress	Stress
Vehicle	7 M, 5F	7 M, 5F	5 M, 5F	5 M, 5F	7 M, 6F	7 M, 6F
CBD	8 M, 5F	7 M, 5F	5 M, 5F	5 M, 5F	8 M, 4F	8 M, 4F
3:1 CBD:THC	10 M, 7F	11 M, 9F	5 M, 5F	5 M, 5F	10 M, 5F	13 M, 5F

M, male; F, female.

### Plasma collection and analysis

For CBD and THC content analysis, only the plasma of mice injected with 3:1 CBD:THC mixture was analyzed. The plasma of a subgroup of mice (*n* = 37) that underwent behavior was collected through tail bleeds at two different time points following the behavior. The first bleed was immediately after the end of the open field test or 80 min post-3:1 CBD:THC injection (T80). The second bleed was 140 min post-injection (T140), equivalent to 60 and 70 min after the end of open field test. To analyze the level of CBD and THC at its expected peak plasma concentration time point, a different group of mice (*n* = 19) were injected with the 3:1 CBD:THC mixture and plasma was collected 45 min after the injection (T45), without any additional behavioral measures.

### Mass spectrometry

Analysis of plasma CBD and ∆^9^-THC levels were performed according to previously described methods (Sepulveda et al., 2022; Carkaci-Salli et al., 2023). Briefly, standard curves were constructed by plotting the ratio of peak area of analyte to peak area of the corresponding internal standard versus analyte concentration. Treated plasma and standards were processed the same way: after spiking 4 μL internal standards into 10 μL plasma, samples were vortexed and 26 μL acetonitrile/H_2_O/formic acid (90/10/0.1) was added for extraction. The calculated concentrations from the standard curves were adjusted by times 4 to reflect the *in vivo* levels of THC and CBD in plasma.

THC and CBD in plasma were analyzed using a Sciex QTRAP 6500+ mass spectrometer equipped with an electrospray ionization probe operated in positive mode and coupled with a Sciex EXion HPLC separation system. A 1.7 μm Acquity UPLC BEH C18 analytical column (2.1 × 100 mm, Waters, Ireland) was used to separate THC and CBD and other isomers as well as impurities. Multiple reaction monitoring mode was used to analyze and quantify THC and CBD, as well as internal standards, with the transitions of m/z 315 > 193 for THC and CBD, and 318 > 196 for standards (THC-d3 and CBD-d3, Cerilliant, a Sigma-Aldrich company, Round Rock, TX). All peaks were integrated and quantified using Sciex OS 1.5 software.

### Immunohistochemistry (IHC)

Mice were anesthetized immediately after the end of the behavioral/plasma assessments using isoflurane (Vedco, AP/DRUGS/220/96) and perfused through the heart with 10 mL 0.1 M phosphor-buffered saline (PBS, diluted from either Sigma-Aldrich, P3619 or Invitrogen, AM9625), followed by 15 mL of 4% paraformaldehyde (Electron Microscopy 15,714-S) in 0.1 M PBS. The brains were excised, post-fixed with 4% paraformaldehyde in 0.1 M PBS overnight at 4°C. Following the fixation, the brains were transferred to 30% sucrose with sodium azide in 0.1 M PBS for at least 3 days at 4°C. Coronal slices of 40 μm thickness containing the CeA were prepared on a cryostat (Leica CM1850). The slices were placed in a cryoprotectant (3:3:2 mixture of glycerol (Thermo Scientific, 158,920,025 or Sigma-Aldrich, G7893), ethylene glycol (VWR International, BDH1125), 0.1 M PBS) for storage prior to IHC staining.

For staining, free-floating slices containing the CeA were washed in 0.1 M PBS for 10 min, four times. The slices were then permeabilized with 0.5% Triton X-100 in PBS (Acros, 327,372,500) for 30 min and blocked with 10% Normal Donkey Serum (Abcam, ab7475, or Jackson ImmunoResearch, 017–000-121) in PBS containing 0.1% Triton X-100 for 1 h. Primary antibodies [Rabbit Anti-Iba-1 (1:1000 dilution; Abcam, ab178846) to assess microglia morphology, or Rabbit Anti-S100β (1:1000 dilution; Abcam, ab52642) to assess astrocytes] were added to the blocking solution in separate analyses. Following primary antibody treatment, the slices were then incubated with primary antibodies/blocking solution for 72 h at 4°C while covered with aluminum foil and placed on a nutating mixer. After 72 h, the slices were washed with PBS four times for 15 min each time. The slices were placed in a mixture of 10% Normal Donkey Serum in PBS containing 0.1% Triton X-100 and secondary antibody. For microglia staining, 1:500 Cy-3 Donkey Anti-Rabbit secondary antibody (Jackson ImmunoResearch, 711–166-152) was used, while for astrocyte staining, 1:500 Alexa-Fluor 488 Donkey Anti-Rabbit (Jackson ImmunoResearch, 711–545-152) secondary antibody was used. The slices were then covered with aluminum foil and incubated on a nutating mixer for 24 h at 4°C. After 24 h, the slices were washed with PBS four times for 10 min each time. The slices were then mounted on slides and dried. Once dry, Prolong Gold mounting media (Invitrogen, P36930) was used for cover slipping. Once dry, clear nail polish was used to seal the edges of the cover slip. The CeA of Rabbit Anti-Iba-1-stained slices were imaged at 20x magnification with 1.3 digital zoom on a Zeiss Axio Examiner.Z1 LSM900 Confocal Microscope, images were tiled, stitched and z-stacked using Zen 3.3 and analyzed using ImageJ software. The images were approximately 60 mm^2^ in size. The CeA of Rabbit Anti-S100β stained slices were imaged at 40x magnification on a Keyence BZ-X710 microscope, z-stacked using BZ-X Analysis software, and analyzed using QuPath-0.4.4. The images were approximately 102 mm^2^ in size. To fully visualize cell bodies, exposure was altered accordingly for each slice. For microglial staining, activated and resting microglia were differentiated based on their morphology as previously described (Coker et al., 2022). Briefly, cells with a small, round cell body with many long and thin processes were considered resting. Cells with larger, oblong or amoeboid cell bodies and with few or shorter processes were considered active. It is important to note that microglia exist in a range of activity states, however, to simplify the quantification of microglial activity, we chose to mark any microglial cells that were not in a clear resting state as active. Since S100β is highly expressed in activated astrocytes (Gonçalves et al., 2008; Bramanti et al., 2010), the number of S100β positive cells was counted and compared between each of the groups as proxy measure for astrocyte activation states.

### Statistics and data analysis

The primary goal of analyses was to compare the mean outcome levels related to various measurements, including time spent in center, distance traveled, time of immobility, and ratio of time spent immobile in the center based on different treatments (i.e., CBD alone or CBD:THC mixture relative to vehicle; alcohol withdrawal timepoints; stress; sex). The measurements were taken at two specific withdrawal time points: 4-and 24-h after the end of CIE. To account for potential confounding factors, the analysis considered the effects of alcohol withdrawal (relative to no alcohol exposure), restraint-induced stress (relative to no restraint-induced stress), and sex (female relative to male). Additionally, the study explored whether the treatments were influenced by interacting factors between treatment and alcohol withdrawal, treatment and stress, and treatment by alcohol by stress. These interaction effects were added to the main effects models for each outcome. With the exception of the plasma concentration analysis, a standard ANOVA was not sufficient for most outcomes. Therefore, for behavioral and immunohistological measures, all models used a negative binomial distribution that was chosen as it was a better model for the dataset compared to the normal or Poisson distributions due to its lower value of the Bayesian Information Criterion (Supplementary Tables 3, 4). There are three critical reasons why the use of the negative binomial distribution and the rate ratios (RRs) that it generates are more useful approaches to analyzing this data than an ANOVA. First, the data is not normally distributed, a key assumption for the correct application of ANOVA. Second, whereas ANOVA becomes cumbersome with more than one or two covariates, regression models provide associations between each explanatory variable and the estimated associations are adjusted for as many covariates as the data can support. Finally, whereas ANOVA does indicate when means are different, it does not provide granular information on how much the outcomes change with incremental change in the explanatory variable after adjustment for covariates. The RRs indicate how much, on average, the count outcome varies with incremental change in each of the explanatory variables. Least square means were also calculated for the outcomes for all combinations of the exposures and any differences among those exposures that were of interest (see supplementary Table 5). The *p*-values of the statistical tests of differences among the least square means generated by each of the interaction models were adjusted for multiple comparisons using a false discovery rate of 5%. SAS version 9.4 was used for these models. BioRender and PowerPoint for Microsoft 360 MSO were used for figure preparation. For plasma concentration analysis, where the number of variables did not require the negative binomial distribution, a t-test and mixed-model ANOVA were used instead. GraphPad Prism 9 was used for the t-test and ANOVA and for figure preparation. Data is represented as mean ± standard error of the mean (SEM). All assessments used a two-tailed p-value of 0.05 to define statistical significance. To demonstrate that the significant associations detected were reasonable for our sample, we conducted a post-hoc power analysis of the significant main effect with the smallest magnitude evaluated in our sample. This was specifically the association of female sex with time in center in the 4-h withdrawal sample which consisted of 86 mice in the control arm and 60 mice in the alcohol arm. Because this association had the smallest magnitude of association that retained statistical significance, by showing that theoretical power calculations based on this data can detect this size effect or smaller, we will provide justification for the observance of all significant associations in our study. Because there are no standard statistical packages that calculate power for the negative binomial distribution, we used a Poisson distribution with overdispersion of 30% to approximate one. This calculation was made using PASS 2023 statistical software and further assumes a Poisson distribution with an overall mean of 21.2 min, power of 80%, and a two-sided alpha of 5%. Outliers were analyzed and removed through GraphPad Prism using the ROUT method with Q = 1%.

## Results

### Behavioral experiments: timing of alcohol withdrawal produces variable effects on anxiety-like behavior in the open field test

To compare the effect of cannabinoids on alcohol-withdrawal induced anxiety, male and female mice were given 8 exposures to binge level alcohol in the CIE model and then examined for CBD and CBD:THC effects on behavior in the open field test at 4- and 24-h withdrawal from CIE. Additional impact of restraint stress prior to behavioral testing was also assessed. A negative binomial distribution was used to model the data comparing time spent in center, time spent immobile, distance traveled, and ratio of time spent immobile in the center of the open field arena across the different alcohol, treatment, stress, and sex conditions. For all negative binomial distribution analyses, the air exposed, vehicle injected, non-stressed male mice were used as a control.

#### CBD decreases time spent in center at 4-h withdrawal, with no effect on 24-h withdrawal, while 3:1 CBD:THC increases time spent in center at 24-h withdrawal, with no effect on 4-h withdrawal

**Figure 2:**
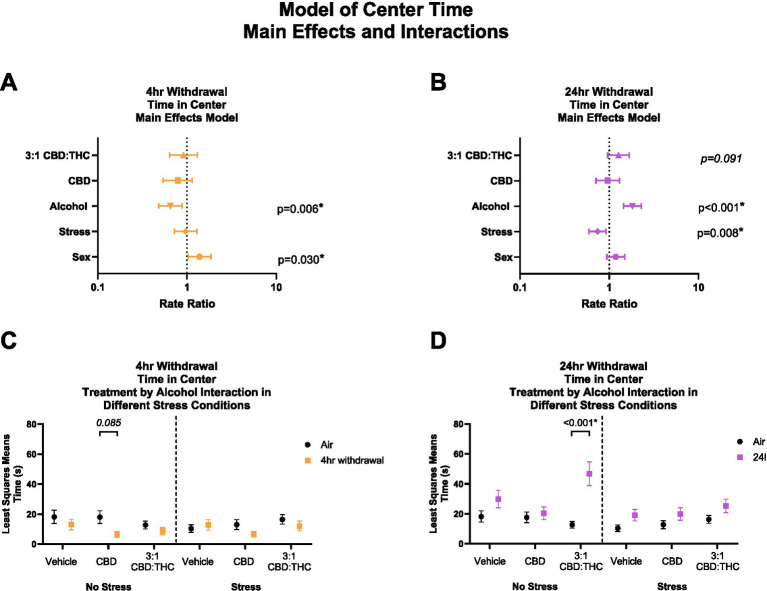
Main effects and interactions model between air control group and each of the withdrawal time groups in the time spent in the center. **(A)** Main effects of treatment, alcohol, stress, and sex in 4-h withdrawal. **(B)** Main effects of treatment, alcohol, stress, and sex in 24-h withdrawal. **(C)** Treatment by alcohol interaction of time spent in the center stratified by stress in 4-h withdrawal. **(D)** Treatment by alcohol interaction of time spent in the center stratified by stress in 24-h withdrawal. The *p*-values represent values of a statistical test of differences between the least square means outcome values for those combinations of explanatory factors. *p* < 0.05 is considered significant.

Figure 2 shows the main effects and interactions of the negative binomial distribution model for the outcome of time spent in the center of the OFT. Generally, in this behavioral paradigm, mice that spend more time in the center are considered to have less anxiety-like behaviors, while mice that spend less time in the center are considered to have more anxiety-like behaviors.

Overall, main effects analysis showed that 4-h withdrawal from CIE (Figure 2A) was associated with reduced time spent in the center (*p* = 0.006), suggesting increased anxiety-like behavior at this time point, while female mice spent more time in the center compared to males (*p* = 0.030). Because this latter association of female sex with time in center among mice with 4 h withdrawal is the smallest magnitude of any main effect association with a significant *p*-value, its empirical values were plugged into statistical software (PASS 2023) to detect the smallest size change observable based on a Poisson distribution with 30% overdispersion, 20% of its variability attributable to covariates, 80% power, and a two sided alpha of 5%. Based on the mean minutes observed for this outcome, the software indicated that we were powered to detect RRs as low as 1.13. Because we detected an RR of 1.18, we have theoretical justification for the observation of this effect in our sample. This suggests that we are also adequately powered to detect the larger effects reported. There was no significant effect of treatment (CBD: *p* = 0.208, 3:1 CBD:THC: *p* = 0.605) or stress (*p* = 0.798). Therefore, mice in 4-h alcohol withdrawal had increased anxiety-like behavior compared to no alcohol mice, with female mice showing less anxiety-like behaviors compared to males and no significant main effects of cannabinoids in reducing anxiety-like behavior in this model.

We then performed a least-squares means interaction outcome analysis for the different combinations of explanatory variables and used multiple comparisons to see the effect of the treatments and stress on time in center. In mice in 4-h withdrawal (Figure 2C) with no additional stressor (Alcohol+No Stress), interaction analysis showed that CBD treatment trended to reduce the time in center compared to air control mice (*p* = 0.085). There were no significant interaction effects in the vehicle or CBD:THC groups in either the no stress or stress conditions. None of the main interaction effects were significant, although Stress x 3:1 CBD:THC treatment interaction approached significance (*p* = 0.071, Supplementary Table 1).

In the 24-h withdrawal condition, mice exposed to the alcohol spent more time in the center compared to air control mice (*p* < 0.001), while mice exposed to the additional stressor spent significantly less time in the center (*p* = 0.008) compared to no stress mice. There was no main effect of sex (*p* = 0.150) or treatment, although 3:1 CBD:THC treatment trended to increase time spent in the center (*p* = 0.091).

In 24-h withdrawal (Figure 2D), interaction analysis showed that 3:1 CBD:THC treatment produced a significant increase in time spent in the center in mice that did not have an additional stressor (Alcohol+No Stress) compared to the same treatment in air control mice (No Alcohol+No Stress, *p* < 0.001). There were no significant differences in the stressed mice. The interaction analysis uncovered significant Alcohol x 3:1 CBD:THC Treatment interaction effect (*p* = 0.033) and a significant Stress x 3:1 CBD:THC Treatment effect (*p* = 0.036) for increased center time. The Alcohol x Stress x 3:1 CBD:THC Treatment interaction approached significance (*p* = 0.068). None of the other interactions were significant (Supplementary Table 2).

Overall, mice in 4-h withdrawal spent less time in the center compared to the controls, while mice in 24-h withdrawal spent more time in the center. Additionally, CBD treatment had a larger effect on mice in 4-h withdrawal, with no effect in 24-h withdrawal. Interestingly, 3:1 CBD:THC treatment significantly increased time spent in the center in non-stressed mice, with no significant effect in stressed mice.

#### 4-H withdrawal decreases distance traveled with CBD and 3:1 CBD:THC having no effect on locomotion

**Figure 3:**
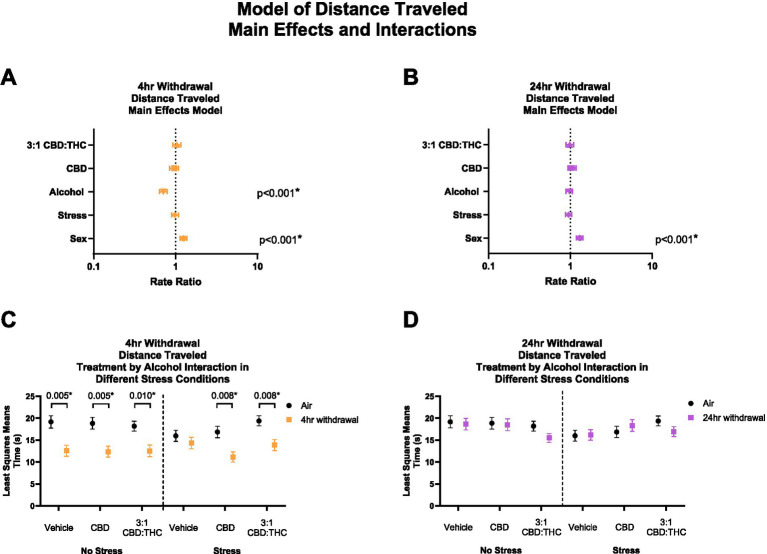
Main effects and interactions model between air control group and each of the withdrawal time groups in total distance traveled. **(A)** Main effects of treatment, alcohol, stress, and sex in 4-h withdrawal. **(B)** Main effects of treatment, alcohol, stress, and sex in 24-h withdrawal. **(C)** Treatment by alcohol interaction total distance traveled stratified by stress in 4-h withdrawal. **(D)** Treatment by alcohol interaction of total distance traveled stratified by stress in 24-h withdrawal. The p-values represent values of a statistical test of differences between the least square means outcome values for those combinations of explanatory factors. *p* < 0.05 is considered significant.

THC and other cannabinoids are known to induce catalepsy and acutely reduce locomotion in animal models, which may confound the results above. We therefore measured total distance traveled in the mice that underwent the behavioral paradigm above. In 4-h withdrawal (Figure 3A), there was no main effect of 3:1 CBD:THC (*p* = 0.531), CBD (*p* = 0.521), or stress (*p* = 0.674). There was a significant effect of alcohol exposure, with mice in 4-h withdrawal traveling significantly less distance (*p* < 0.001). There was also a significant effect of sex, with females traveling more distance (*p* < 0.001). Through the least squares means analysis, we found that mice in 4-h withdrawal (Alcohol) traveled less distance in all groups, except for vehicle stress compared to air control (No Alcohol; Figure 3C). There were no significant interaction effects on any measure (Supplementary Table 1).

In 24-h withdrawal, the main effects analysis did not show significant effect of treatment, alcohol exposure, or stress on the distance traveled (Figure 3B). Similar to 4-h withdrawal, there was a significant effect of sex (*p* < 0.001) in the 24-h withdrawal condition, with female mice traveling more distance than males. There were no significant differences between air control (No Alcohol) and 24-h withdrawal (Alcohol) mice in terms of treatment and stress conditions in the least squares means interaction analysis corrected for multiple comparisons (Figure 3D). None of the interaction effects were significant (Supplementary Table 2).

Overall, these results show decreased distance traveled in 4-h but not 24-h CIE withdrawal groups compared to control, but no effects of CBD or CBD:THC treatments on distance traveled suggesting limited cannabinoid tetrad effects that could confound results in this model.

#### 3:1 CBD:THC and 4-h withdrawal increase total time spent immobile

**Figure 4:**
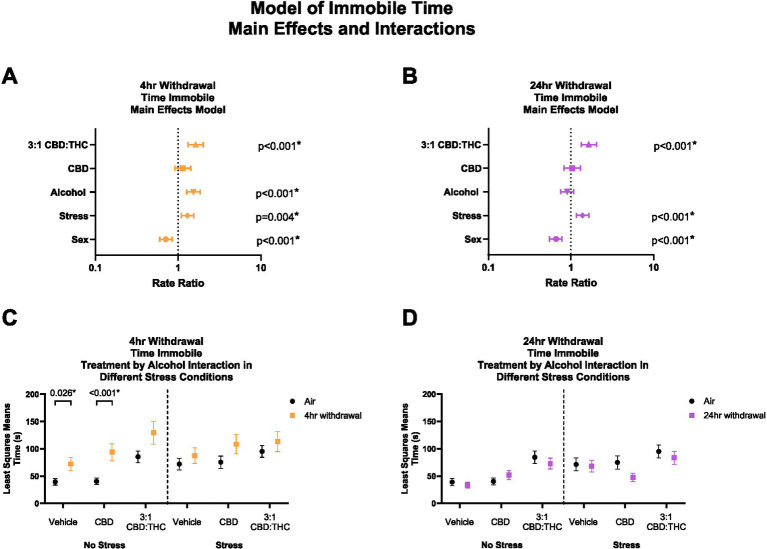
Main effects and interactions model between air control group and each of the withdrawal time groups in the time spent immobile. **(A)** Main effects of treatment, alcohol, stress, and sex in 4-h withdrawal. **(B)** Main effects of treatment, alcohol, stress, and sex in 24-h withdrawal. **(C)** Treatment by alcohol interaction of time spent immobile stratified by stress in 4-h withdrawal. **(D)** Treatment by alcohol interaction of time spent immobile stratified by stress in 24-h withdrawal. The p-values represent values of a statistical test of differences between the least square means outcome values for those combinations of explanatory factors. *p* < 0.05 is considered significant.

Time immobile in the OFT can be used to further assess anxiety-like behaviors, whereby increased immobility indicates an increase in anxiety-like behaviors. Given that total distance travelled was not altered by CBD:THC, we next looked at the total time mice spent immobile in the OFT. In 4-h withdrawal (Figure 4A), there was a significant main effect increase in time immobile by alcohol exposure (*p* < 0.001), stress exposure (*p* = 0.004), and 3:1 CBD:THC treatment (*p* < 0.001) compared to controls. Female mice spent less time immobile (*p* < 0.001). CBD treatment did not lead to any changes in time immobile (*p* = 0.267).

We then performed a least squares means interaction outcome analysis for the different combinations of explanatory variables correcting for multiple comparisons to see the effect of the treatments and stress on immobility time. In 4-h withdrawal, we observed increased time spent immobile in the Vehicle (*p* = 0.026) and CBD (*p* < 0.001) groups in the no stress condition (Alcohol+No Stress) compared to air control (No Alcohol+No Stress; Figure 4C). However, after stress exposure (Alcohol+Stress), none of the treatment groups had significant changes from air control (No Alcohol+Stress). It is important to note that basal time spent immobile in non-stressed air control (No Alcohol+No Stress) mice is lower than in stressed air control mice (No Alcohol+Stress). Therefore, the lack of treatment effects in the 4-h withdrawal group might be due to a higher baseline of time spent immobile at 4-h withdrawal and a ceiling effect after the treatments (Figure 4C). There were no significant main interaction effects, although Stress × 3:1 CBD:THC Treatment interaction approached significance (*p* = 0.074, Supplementary Table 1).

In 24-h withdrawal (Figure 4B), main effects analysis showed that 3:1 CBD:THC treatment (*p* < 0.001) and additional stress exposure (*p* < 0.001) significantly increased time spent immobile compared to controls. Females spent less time immobile (*p* < 0.001). There were no interaction differences in time spent immobile in the no stress or the stress 24-h withdrawal group (Alcohol+No Stress) compared to control (No Alcohol+No Stress) in any of the treatments (Figure 4D). There were no significant main interaction effects in 24-h withdrawal, although Alcohol x Stress x CBD Treatment approached significance (*p* = 0.076, Supplementary Table 2).

Overall, these data indicate that stress exposure increases time spent immobile in both withdrawal conditions, and mice are more sensitive to the anxiogenic effects of alcohol withdrawal at the 4 h timepoint. Additionally, female sex was protective against stress, as seen by reduced time immobile in both withdrawal groups.

#### 3:1 CBD:THC increases ratio of time spent immobile in center at 24-h withdrawal

Typically, increases in immobile time are thought to indicate increases in anxiety-like behaviors in the open field test. However, in our data we saw a significant increase in immobility time in mice treated with 3:1 CBD:THC, and a trending increase in time spent in the center in 24-h withdrawal mice, suggesting that some immobility time could have occurred in the center zone, an atypical behavior in this model. Therefore, we next sought to measure the ratio of time the mice spent immobile in the center relative to total center time to further examine anxiety-like behaviors in this study.

**Figure 5:**
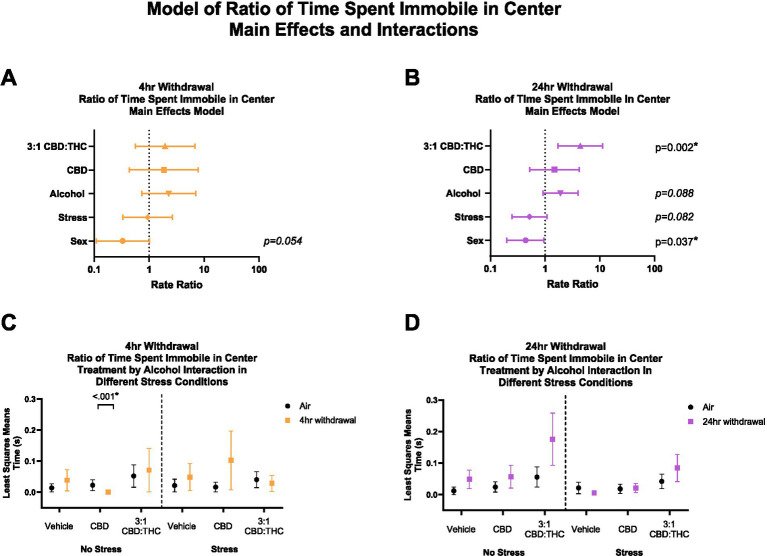
Main effects and interactions model between air control group and each of the withdrawal time groups in ratio of time spent immobile in the center. **(A)** Main effects of treatment, alcohol, stress, and sex in 4-h withdrawal. **(B)** Main effects of treatment, alcohol, stress, and sex in 24-h withdrawal. **(C)** Treatment by alcohol interaction of ratio of time spent immobile in the center stratified by stress in 4-h withdrawal. **(D)** Treatment by alcohol interaction of ratio of time spent immobile in the center stratified by stress in 24-h withdrawal. The p-values represent values of a statistical test of differences between the least square means outcome values for those combinations of explanatory factors. *p* < 0.05 is considered significant.

For this analysis, 7 mice were excluded from analysis (2 in the air control group and 5 in the 4-h withdrawal group) as these mice did not spend time in the center. In 4-h withdrawal (Figures 5A,C), there were no significant main effects. Interaction analysis showed that CBD injected mice not exposed to a stressor (Alcohol+No Stress) had a significant reduction in the ratio of time spent immobile in the center compared to air controls (No Alcohol+No Stress; *p* < 0.001). It is worth noting that all the mice in the CBD no-stress group spent none of their time in the center as immobile and, therefore, have no variability in their data. There were no other significant effects of 4-h withdrawal compared to air control in either stress or treatment conditions. There was a significant main interaction effect of Alcohol x CBD treatment (*p* < 0.001) in 4-h withdrawal, with no other significant interaction effects (Supplementary Table 1).

Main effects analysis found a significantly increased ratio of time spent immobile in the center in 24-h withdrawal (Figures 5B,D) mice exposed to 3:1 CBD:THC (*p* = 0.002), when controlled for stress, sex, and alcohol exposure. There was a significant main effect of sex in 24-h withdrawal (*p* = 0.037), with females spending less time immobile in the center. There were no significant interaction effects (Supplementary Table 2).

Overall, these data suggest that mice treated with 3:1 CBD:THC spend a higher proportion of their time in the center in a state of immobility in 24-h but not 4-h withdrawal, which may indicate a distinct mechanism of anxiolysis in this group.

### Plasma analysis: 24-H withdrawal mice have increased THC content and metabolize CBD faster than mice in the air group

**Figure 6:**
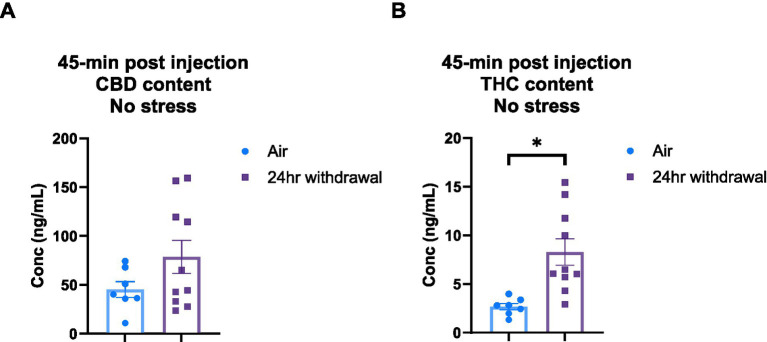
Plasma content of CBD and THC in air and 24-h withdrawal groups at 45 min post-injection. **(A)** CBD content 45 min post-injection. **(B)** THC content 45 min post-injection. Note the difference in y axis between **A** and **B**. None of the mice had the additional stressor at the time of blood collection. *represents significant group difference between air and 24-h withdrawal groups. Statistical outliers were removed from the analysis. *p* < 0.05 was considered significant.

It is widely understood that chronic alcohol exposure like CIE can cause damage to end organs, including the liver (Mouton et al., 2016). Since both CBD and THC are metabolized in the liver (Zhu and Peltekian, 2019), we wanted to explore the effect of the chronic alcohol exposure on CBD and THC metabolism. Therefore, we performed a THC and CBD plasma content analysis in air control and 24-h withdrawal mice injected with 3:1 CBD:THC. The plasma was analyzed at 45-, 80-, and 140-min after the injection of 3:1 CBD:THC. Mass spectrometry analysis of the plasma at 45 min after the injection showed no significant differences in CBD concentration between air and withdrawal mice (*p* = 0.142, *t* = 1.552, df = 15, Figure 6A), but significantly increased THC concentration (*p* = 0.004, *t* = 3.382, df = 15, Figure 6B).

**Figure 7:**
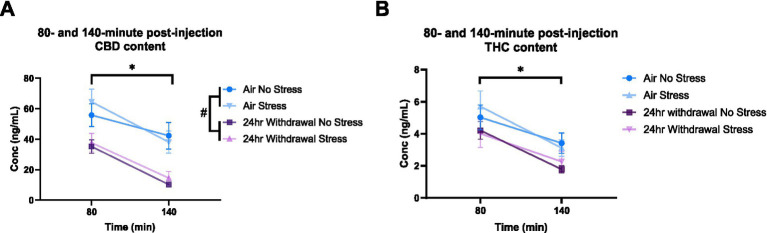
Plasma content of CBD and THC in air and 24-h withdrawal groups at 80- and 140-min post-injection. **(A)** CBD content at 80- and 140-min post-injection. **(B)** THC content at 80- and 140-min post-injection. Both no stress and stress groups were included in the analysis. *represents significant main effect of time between time point 80 and time point 140. #represents significant main effect of alcohol. Statistical outliers were removed from the analysis. *p* < 0.05 was considered significant.

At 80- and 140-min post-injection, mixed-model ANOVA for CBD concentration (Figure 7A) showed a significant effect of alcohol exposure (*p* < 0.001, *F*_(1,31)_ = 19.27) and a significant effect of time (*p* < 0.001, *F*_(1,30)_ = 70.76). There were no significant effects of stress or any interaction effects. Sidak’s multiple comparisons examining differences between the 80- and 140-min timepoints within each group showed that air control mice that were not exposed to a stressor did not have changes in CBD (*p* = 0.193) content between the two timepoints. Air stress (*p* < 0.001), 24-h withdrawal no stress (*p* < 0.001), and 24-h withdrawal stress (*p* < 0.001) groups had significant reductions in CBD content between 80 and 140 min. Multiple comparisons test examining the differences between groups at each time point, found a significant difference between the air no stress and the 24-h withdrawal no stress groups at 140 min (*p* = 0.005).

For THC content (Figure 7B), mixed-model ANOVA showed a significant effect of time (*p* < 0.001, *F*_(1,33)_ = 71.44), with no significant effects on any of the other measures, although alcohol exposure trended to approach significance (*p* = 0.068, F_(1,33)_ = 3.569). The mixed-model ANOVA showed no significant interaction effects. Sidak’s multiple comparisons exploring the effect of time for each group showed significant differences between the 80- and 140-min timepoints in all conditions (air no stress, *p* = 0.043; air stress, *p* < 0.001; 24-h withdrawal no stress, *p* < 0.001; 24-h withdrawal stress, *p* = 0.004).

Overall, these results show elevated THC concentrations at expected peak time points and faster metabolism of CBD in mice exposed to 24-h withdrawal compared to control mice. This difference in THC content and CBD metabolism may be related to the increased immobility of 24-h withdrawal mice.

### Immunohistochemistry: decreased number of S100β-positive astrocytes and microglial cell density in 4-h, but not 24-h, withdrawal

#### 4-h withdrawal decreases the number of astrocytes in the CeA

**Figure 8:**
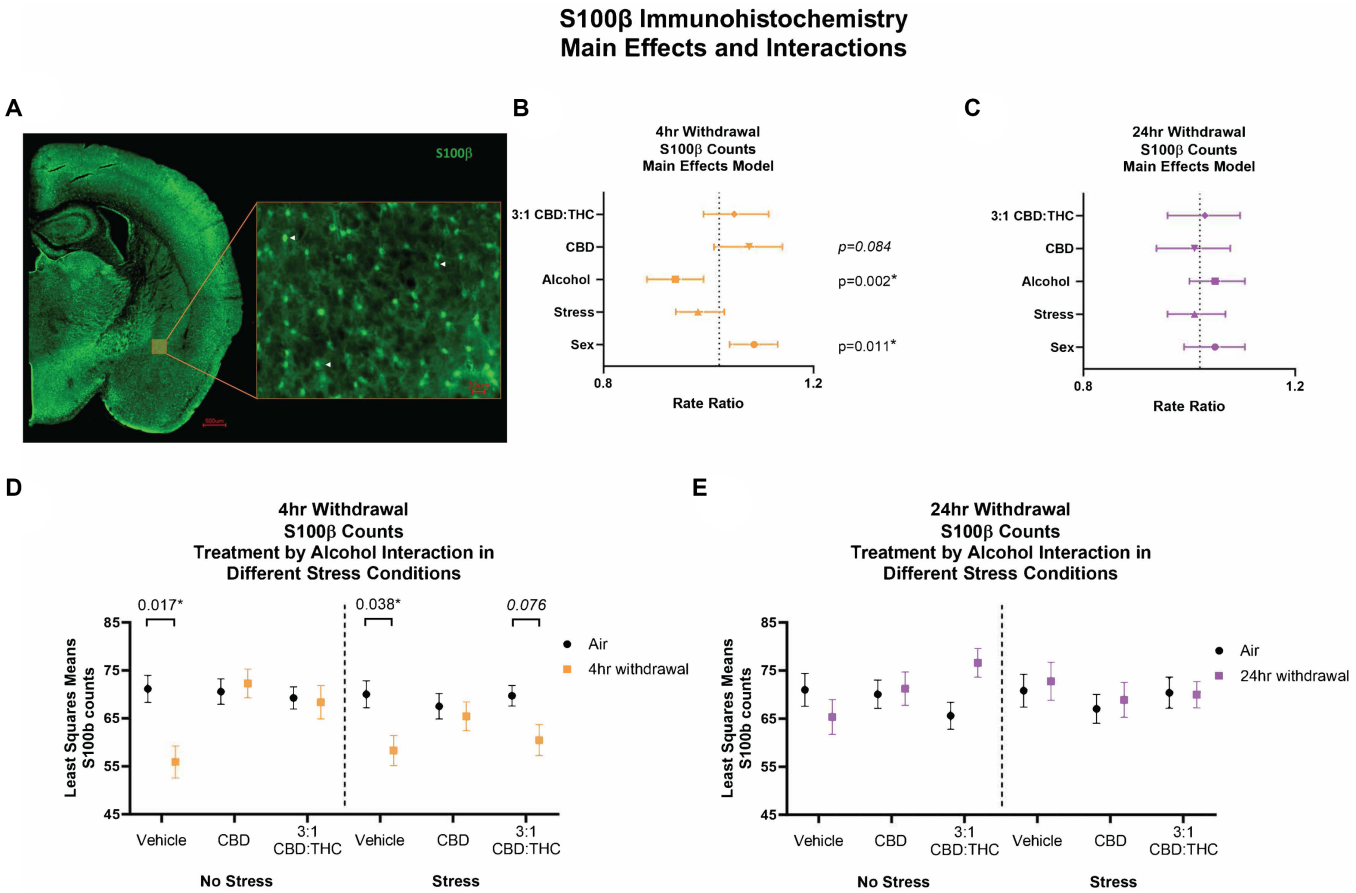
Main effects and interactions model between air control group and each of the withdrawal time groups in the number of S100β + cells. **(A)** Representative image of CeA location and S100β + cells. White arrows point to examples of counted cells. **(B)** Main effects of treatment, alcohol, stress, and sex in 4-h withdrawal. The x-axis on a log_10_ scale has been modified to better visualize the data. **(C)** Main effects of treatment, alcohol, stress, and sex in 24-h withdrawal. The x-axis on a log_10_ scale has been modified to better visualize the data. **(D)** Treatment by alcohol interaction of the number of S100β+ cells stratified by stress in 4-h withdrawal. **(E)** Treatment by alcohol interaction of the number of S100β+ cells stratified by stress in 24-h withdrawal. The *p*-values represent values of a statistical test of differences between the least square means outcome values for those combinations of explanatory factors. *p* < 0.05 is considered significant. Data is collected from at least 6 mice per group and is averaged across both hemispheres between 2 brains slices per mouse.

We counted the number of S100β-positive astrocytes as a proxy measure of activated astrocytes in the CeA (Figure 8A). Following 4-h withdrawal, there was a significant effect of alcohol (*p* = 0.002), with reduced numbers of S100β + cells (Figure 8B). There was also a significant sex effect, with females having an increase in the number of S100β + cells (*p* = 0.011). There were no other significant main effects. After 24-h withdrawal, there was no significant main effect in any of the measures (Figure 8C).

We then performed a least squares means interactions with multiple comparisons analysis comparing each of the withdrawal conditions to air control. In 4-h withdrawal (Figure 8D), vehicle injected mice in the no stress condition (Alcohol+No Stress) had reduced numbers of S100β + cells compared to air control (No Alcohol+No Stress; *p* = 0.017). There were no differences in CBD (*p* = 0.844) or 3:1 CBD:THC (*p* = 0.899) injected groups, suggesting these cannabinoids may have restored disrupted astrocyte activation states at this withdrawal time point. In the stress condition (Alcohol+Stress), the vehicle group once again had significantly reduced number of S100β + cells following 4-h withdrawal compared to air control (No Alcohol+Stress; *p* = 0.038). Mice injected with 3:1 CBD:THC (*p* = 0.076) had reduced numbers of S100β + cells when in 4-h withdrawal compared to air control. The CBD injected groups had no differences between 4-h withdrawal and air control (*p* = 0.811). There was a significant Alcohol x CBD treatment (*p* = 0.003) and Alcohol x 3:1 CBD:THC treatment (*p* = 0.015) interaction effect, showing an increase in the cell numbers after the CBD and the 3:1 CBD:THC treatments in the 4-h withdrawal group compared to vehicle treatment (Supplementary Table 1).

After 24-h withdrawal (Figures 8C,E), there were no significant main effects of stress or alcohol exposure. There was a significant Alcohol x 3:1 CBD:THC treatment effect showing an increase in the S100β + cell numbers compared to no alcohol vehicle exposure treatment (Supplementary Table 2).

#### 4-H withdrawal decreases microglia density in the CeA

**Figure 9:**
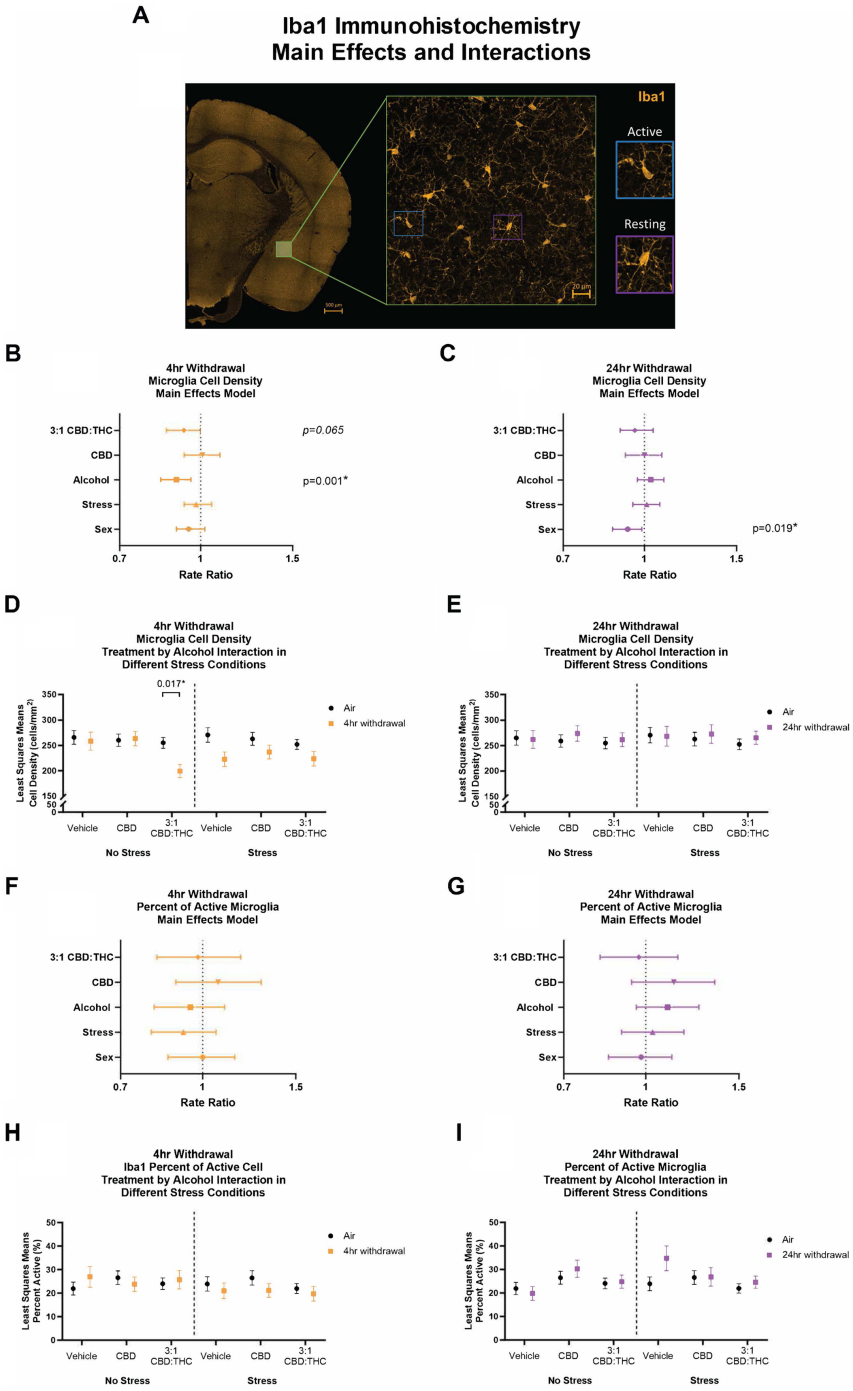
Main effects and interactions model between air control group and each of the withdrawal time groups in the density of Iba1+ microglial cells and the percent of active microglial cells. **(A)** Representative image of CeA location and Iba1+ cells. Examples of active and resting microglia are portrayed on the right side of the image. **(B)** Main effects of microglial cell density for treatment, alcohol, stress, and sex in 4-h withdrawal. The x-axis on a log_10_ scale has been modified to better visualize the data. **(C)** Main effects of microglial cell density for treatment, alcohol, stress, and sex in 24-h withdrawal. The x-axis on a log_10_ scale has been modified to better visualize the data. **(D)** Treatment by alcohol interaction of microglial cell density stratified by stress in 4-h withdrawal. **(E)** Treatment by alcohol interaction of microglial cell density stratified by stress in 24-h withdrawal. **(F)** Main effects of percent of active microglia for treatment, alcohol, stress, and sex in 4-h withdrawal. The x-axis on a log_10_ scale has been modified to better visualize the data. **(G)** Main effects of percent of active microglia for treatment, alcohol, stress, and sex in 24-h withdrawal. The x-axis on a log_10_ scale has been modified to better visualize the data. **(H)** Treatment by alcohol interaction of percent of active microglial cells stratified by stress in 4-h withdrawal. **(I)** Treatment by alcohol interaction of percent of active microglial cells stratified by stress in 24-h withdrawal. The p-values represent values of a statistical test of differences between the least square means outcome values for those combinations of explanatory factors. *p* < 0.05 is considered significant. Data is collected from at least 6 mice per group and is averaged across both hemispheres between 2 brains slices per mouse.

We next stained CeA sections for Iba1, a microglial-specific marker that allows for analysis of overall cell numbers, as well as microglia morphology to assess activation states (Lier et al., 2021; Coker et al., 2022). An experimenter, blinded to the conditions, quantified the density and percentage of active microglia (Figure 9).

After 4-h withdrawal, main effect analysis found a reduction in microglial cell density (cells/mm^2^, *p* = 0.001). None of the other main effects were significant, although 3:1 CBD:THC trended to reduce microglial cell density (*p* = 0.065). When we conducted a least squares means interaction analysis, we found that, in the no stress condition, 3:1 CBD:THC treatment reduced the microglial cell density in the 4-h withdrawal group (Alcohol+No Stress) compared to the air mice (No Alcohol+No Stress; *p* = 0.017). There were no differences in the vehicle or the CBD injected mice. In the stress condition, there were no significant differences. None of the interactions were significant; however, Alcohol x 3:1 CBD:THC treatment approached significance (*p* = 0.057, Supplementary Table 1).

In the 24-h withdrawal group, there was no significant main effect of alcohol, treatment, or stress; however, there was a significant main sex effect, with females having a reduced microglial cell density (*p* = 0.019). Through the least squares means interaction analysis, we found no significant differences between the 24-h and the air control groups in any treatment or stress conditions. There were also no significant interaction effects (Supplementary Table 2).

## Discussion

The goal of this study was to assess the role of cannabinoids in alcohol withdrawal-induced anxiety-like behaviors and determine if this is correlated to changes in CeA neuroimmune cell activation states. In terms of anxiety-like behaviors we hypothesized that (1) alcohol withdrawal will increase anxiety-like behavior; (2) an acute stressor will exacerbate alcohol-withdrawal induced anxiety-like behaviors; and (3) both the 3:1 CBD:THC mixture and CBD alone will significantly reduce anxiety-like behaviors during withdrawal. Supporting our hypotheses, our findings showed increased baseline anxiety at 4-h withdrawal and that an additional acute stressor increased anxiety-like behaviors at 24-h withdrawal. Our hypothesis that CBD and 3:1 CBD:THC will decrease anxiety was not fully supported, with increased anxiety at 4-h withdrawal and decreased anxiety at 24-h withdrawal, which may be driven by the altered metabolism of CBD and THC at these withdrawal timepoints. We further hypothesized that alcohol withdrawal will increase S100β cell counts and alter Iba1 morphology and density in the CeA, suggestive of increased neuroinflammation by alcohol as previously shown (Melkumyan frontiers review). We further expected alcohol effects on S100β and Iba1 to be attenuated by CBD and 3:1 CBD:THC administration. Surprisingly, we found decreased S100β cell counts and altered Iba1 markers after alcohol withdrawal, particularly at 4-h withdrawal, but did find that CBD and 3:1 CBD:THC normalized these changes induced by alcohol-withdrawal.

Alcohol withdrawal is thought to increase sensitivity to stress. Data in our model support these findings as we found that an acute stressor increased anxiety-like behavior in both 4- and 24-h withdrawal as evidenced by increased immobility time in both withdrawal time points (*p* < 0.001 for 24-h and *p* = 0.004 for 4-h) and decreased time in the center for the 24-h withdrawal time point (*p* = 0.008). Our data also showed potential differences in the anxiety-like behaviors between 4- and 24-h withdrawal which may be due to the physiological response to the withdrawal at each of these timepoints. In mice, somatic alcohol withdrawal effects, such as handling-induced convulsions, peak between 4 and 6 h into withdrawal, and these symptoms decrease by 24-h (Olsen et al., 2019) while other work shows extended withdrawal may be needed for psychological symptoms to fully emerge. This difference in withdrawal symptom onset may, in part, also explain the decreased distance traveled by mice in 4-h, but not 24-h, withdrawal.

In terms of cannabinoid treatment, we found that CBD may be anxiogenic in acute alcohol withdrawal compared to air controls, a finding contrary to our initial hypothesis. CBD injected mice in 4-h withdrawal spent less time in the center (*p* = 0.085) and more time immobile (*p* = 0.006) compared to controls. There were no significant effects of CBD in 24-h withdrawal. Therefore, our data suggest that CBD does not reduce anxiety-like behaviors in 4- or 24-h withdrawal and may be anxiogenic in 4-h withdrawal. This finding contradicts the literature showing that CBD reduces or does not affect anxiety at varying doses (Sharpe et al., 2020). This contradiction may be due to the extra variable of alcohol withdrawal, as to our knowledge, no previous studies have examined the role of CBD on alcohol withdrawal-induced anxiety using the vapor alcohol administration model. Other work (Gasparyan et al., 2023) shows that: (1) 40 mg/kg CBD can reduce anxiety-like behaviors in the light–dark test at a 6-h spontaneous withdrawal timepoint following 15 days of increasing alcohol concentrations via oral gavage; (2) repeated 60 mg/kg CBD treatments can ameliorate social stress coping in the resident-intruder paradigm following adolescent alcohol exposure and withdrawal and promotes synaptic plasticity in the nucleus accumbens in rats after oral gavage (Brancato et al., 2023), and (3) repeated 15-30 mg/kg CBD can reduce context- and stress-induced reinstatement of alcohol seeking and increase time in the open arms of an elevated plus maze in rats after oral self-administration (Gonzalez-Cuevas et al., 2018). Overall, more studies are needed to explore the mechanisms underlying these effects of CBD in the context of alcohol withdrawal-induced anxiety and in multiple models of alcohol exposure.

With 3:1 CBD:THC treatments, we surprisingly found increased immobility time in mice in alcohol withdrawal (*p* < 0.001), with larger effects in 24-h withdrawal. We found that mice in 24-h withdrawal injected with 3:1 CBD:THC, particularly in the no stress group, spent significantly more time spent in the center (*p* < 0.001). We hypothesize that this increase in immobility time and the unusual behavior of spending a significant amount of time immobile in the center may be due to effects of THC. THC has a high affinity for cannabinoid receptors 1 and 2 (CB_1_ and CB_2_) and elicits its psychotropic and behavioral effects via activation of the CB_1_ receptor (Tseng and Craft, 2004). THC alone has dose dependent effects on anxiety. Lower doses of THC have been shown to be more anxiolytic, reducing reactivity of the amygdala, while higher doses are linked to increased anxiety and increased amygdala activation (Phan et al., 2008; Bhattacharyya et al., 2017; Childs et al., 2017; Raymundi et al., 2020). This increase in anxiety due to high THC dose is associated with the increased activation of CB_1_ receptors in the amygdala (Bhattacharyya et al., 2017; Childs et al., 2017), although further studies are needed to confirm this finding. CBD has been shown to attenuate and prevent the anxiogenic action produced by higher THC doses (Bhattacharyya et al., 2010; Freeman et al., 2019; Raymundi et al., 2020); although findings are mixed with some studies showing an anxiogenic effect of CBD-THC mixtures (Kasten et al., 2019). It is hypothesized that CBD affects THC by acting on the CB_1_ receptor as a negative allosteric modulator (Laprairie et al., 2015). However, it should be noted that some studies show no effect of CBD on the CB_1_ receptor, while others suggest that CBD indirectly mediates agonism and antagonism of the CB_1_ receptor (Zlebnik and Cheer, 2016).

Increased immobility time in the center is a non-typical outcome in the open field test. Normally, increased immobility in the open field test would suggest increased anxiety-like behavior. However, due to the increase in immobility time occurring in the center and a trending increase in total time spent in the center, it is possible that 3:1 CBD:THC may be reducing anxiety-like behaviors To further interpret the finding of increase immobility in center after 3:1 CBD:THC treatment, it is important to note that 3:1 CBD:THC did not affect overall distance traveled in either withdrawal time point, indicating the increased immobility time is not due to overall decreases in locomotor activity. We hypothesized that this unique outcome may be due to alterations in CBD or THC pharmacokinetics by alcohol exposures. While mechanisms for differential cannabinoid pharmacokinetics after alcohol exposure have not been extensively examined previously, alcohol is well known to negatively impact liver function, leading to changes in hepatic metabolism. At 24-h alcohol withdrawal, it is known that the activity of cytochrome P-450 (CYP450) enzymes in the liver are altered (Roberts et al., 1995). CBD and THC are metabolized through liver CYP450 enzyme pathways, one of which is CYP3A4 (Zhu and Peltekian, 2019). Therefore, it is possible that CBD was preferentially metabolized over THC in alcohol withdrawal compared to air control due to altered liver metabolism processes.

To test this hypothesis, we collected plasma from mice that were injected with 3:1 CBD:THC and were either air control or in 24-h withdrawal. The plasma analysis at 45-min post injection showed increased concentrations of THC in mice in 24-h withdrawal compared to air controls. There was no significant difference in the concentration of CBD. This finding suggests that the pharmacokinetics and/or pharmacodynamics of THC may be altered in mice in 24-h withdrawal compared to air control mice. Further analysis at 80 and 140 min showed that mice in 24-h withdrawal had a reduced CBD content, with no differences in THC content. This reduced CBD content could mean that mice in 24-h withdrawal preferentially metabolize CBD over THC compared to air control mice. It is important to note that we cannot directly compare the data collected at 45 min to the 80- and 140-min timepoints as different sets of mice were used for these studies to avoid additional stress variables in the behavioral paradigm. Additional studies will be necessary to fully determine the pharmacokinetics and pharmacodynamics of CBD and THC metabolism in various alcohol withdrawal conditions and routes of administration. Such work would have extensive public health relevance given the common availability of alcohol and increased cannabis usage in recent years.

We have previously found that alcohol’s actions on the excitatory neurotransmission in the lateral subdivision of the CeA are mediated by astrocytes (Melkumyan et al., 2022), a mechanism that we propose to be driving enhanced alcohol intake and alcohol-related changed in anxiety-like behaviors following chronic alcohol intake (Melkumyan and Silberman, 2022). Contrary to our initial hypothesis that S100β and Iba1 changes would indicate enhanced neuroimmune function during alcohol withdrawal, we found decreased S100β cell counts (*p* = 0.002) and decreased Iba1 cell density (*p* = 0.001) in 4-h, but not 24-h, withdrawal conditions, suggesting reductions in neuroimmune cell activation. While most studies indicate increased activity of neuroimmune cells following chronic alcohol exposure (Crews et al., 2004; Adams et al., 2023), the timing of neuroimmune analysis can impact these findings. For instance, a study showed that rats in 4-h withdrawal following a 35-day liquid alcohol diet exposure had either no changes in gene expression compared to control group or a reduced expression of some surrogate markers of neuroimmune activation, especially compared to 48-h withdrawal (Freeman et al., 2012), supporting our findings in the current study. Regardless of direction of change on S100β and Iba1 analyses due to alcohol exposure at 4-h withdrawal, we found that CBD and 3:1 CBD:THC may normalize alcohol-induced changes in S100β and Iba1. In the S100β analysis, we found a reduced number of S100β-positive cells in the no-stress vehicle group in 4-h withdrawal compared to air control (*p* = 0.017), but this reduction was normalized by both CBD and 3:1 CBD:THC treatment. In the stress condition, CBD alone was more effective at normalizing the (Muramatsu et al., 2004; Raponi et al., 2007; Imbe et al., 2013) β cell counts (Muramatsu et al., 2004; Raponi et al., 2007; Imbe et al., 2013). β cell counts via immunohistochemistry have been used extensively in the previous literature to estimate activation states in the manner performed here (Muramatsu et al., 2004; Raponi et al., 2007; Imbe et al., 2013). However, it should be noted that S100β can be expressed in lower concentrations in non-stimulated astrocytes and that S100β concentrations increase in response to pro-inflammatory stimuli. Future experiments will be needed to confirm that the changes in S100β cell counts in this study are associated with increased S100β concentrations or astrocyte activity states. In microglial density analysis, we saw that 3:1 CBD:THC further reduced the microglial cell density in non-stressed mice in 4-h withdrawal compared to air control (*p* = 0.017), pointing to potential anti-inflammatory effects of the CBD:THC mixture. In the stress condition, we saw that both CBD and 3:1 CBD:THC were effective at normalizing the microglial cell density at 4-h withdrawal. A limitation of this study is the correlative nature regarding S100β and Iba1 immunohistochemistry compared with the behavioral outcomes and the use of one immunohistochemical measure for astrocyte and microglia analysis. Future studies will be needed to modulate and assess neuroimmune cell function more directly during the behavioral tasks and in *post-hoc* analyses to confirm and extend the findings here.

In general, studies on the effect of the CBD:THC mixtures on neuroinflammatory activity are limited, with no studies previously examining these effects in the CeA or in the context of alcohol withdrawal. In the prefrontal cortex, chronic injection of a mixture of high CBD (5 mg/kg)/low THC (0.15 mg/kg) in adolescence resulted in no changes in microglial morphology in adulthood compared to control (Gabaglio et al., 2021). However, THC alone and high THC/low CBD mixtures lead to increased microglial activation. In this study, we did not explore the effect of THC alone or a high THC/low CBD mixture due to pilot studies showing extremely reduced locomotor activity, rendering the OFT paradigm insufficient (data not shown). However, findings in the literature have shown that a dose of 2.5 mg/kg THC is not anxiolytic (Valjent et al., 2002) and may be anxiogenic (Schramm-Sapyta et al., 2007). Thus, combining CBD with low dosage THC may be advantageous over THC-only therapies for reduction of anxiety-like behaviors. The current studies also point to cannabinoid modulation of alcohol-induced neuroimmune dysfunction may be dependent on numerous factors including withdrawal state and cannabinoids used. To understand the role of the CeA neuroimmune system in this acute withdrawal state, further studies are needed to directly modulate CeA neuroimmune systems to affect alcohol-withdrawal induced anxiety. While this study focused on the CeA, additional studies are needed to determine the impact of cannabinoids on neuroimmune function in other relevant brain regions in the context alcohol use disorder models.

Overall, the findings of this study suggest that mice in 4-h withdrawal have increased anxiety, and this increased anxiety is exacerbated by further stressors and cannabinoid treatment. At 24-h of withdrawal there is an altered metabolism of CBD:THC, potentially leading to an increase in relative efficacy of THC due to faster elimination of CBD. Additionally, there is a reduction in microglial density and astrocytic activation at 4-h, but not 24-h withdrawal, highlighting the importance of further studying the neuroimmune activity at these early withdrawal timepoints. Parsing out the mechanism of this neuroimmune activity in alcohol withdrawal in response to cannabinoids will be an important avenue of research as medical and recreational cannabis consumption become more widely available.

## Data availability statement

The original contributions presented in the study are included in the article/Supplementary material, further inquiries can be directed to the corresponding author.

## Ethics statement

The animal study was approved by Penn State College of Medicine Institutional Animal Care and Use Committee. The study was conducted in accordance with the local legislation and institutional requirements.

## Author contributions

MM: Conceptualization, Data curation, Formal analysis, Funding acquisition, Investigation, Methodology, Project administration, Resources, Software, Validation, Visualization, Writing – original draft, Writing – review & editing. VA: Formal analysis, Methodology, Writing – review & editing, Data curation, Investigation. AE: Formal analysis, Methodology, Visualization, Writing – review & editing. OS: Formal analysis, Methodology, Writing – review & editing. ZM: Methodology, Writing – review & editing. DS: Formal analysis, Resources, Software, Writing – review & editing, Data curation, Investigation, Methodology. TM: Data curation, Formal analysis, Visualization, Writing – review & editing, Supervision. KV: Conceptualization, Funding acquisition, Methodology, Resources, Supervision, Writing – review & editing. AA: Conceptualization, Resources, Writing – review & editing. WR-K: Conceptualization, Data curation, Methodology, Resources, Writing – review & editing, Validation. YS: Conceptualization, Data curation, Formal analysis, Funding acquisition, Investigation, Methodology, Project administration, Resources, Software, Supervision, Validation, Visualization, Writing – review & editing.
