# Supplementary material for: Effects of cannabidiol, with and without ∆9-tetrahydrocannabinol, on anxiety-like behavior following alcohol withdrawal in mice
Source: Front Neurosci. 2024 Jun 18;18:1375440. doi: 10.3389/fnins.2024.1375440 (PMC11217543; doi:10.3389/fnins.2024.1375440)
Supplement: Supplementary file 1 [file Data_Sheet_1.docx]

**Supplemental Information**

Supplemental Table 1– Interaction effects of each of the outcomes in 4-hour withdrawal. The estimate values provide the effect size of the interactions. Wald Chi-Square test shows that the value in the estimate column, given the value in the Standard Error column, is different from 0. The p-value represents the significance of the Wald Chi-Square value. Bolded p-values with an asterisk show significant p-values, italicized p-values show trends of significance, although they do not approach significance.

| **4-hour withdrawal Interaction Effects** | | | | |
| --- | --- | --- | --- | --- |
| **Parameter** | **Estimate** | **Standard Error** | **Wald Chi-Square** | **P-Value** |
| ***Time in Center*** | | | | |
| Alcohol x CBD Treatment | -0.6954 | 0.5220 | 1.77 | 0.1828 |
| Alcohol x 3:1 CBD:THC Treatment | -0.0522 | 0.5058 | 0.01 | 0.9178 |
| Stress x CBD Treatment | 0.2370 | 0.4913 | 0.23 | 0.6295 |
| Stress x 3:1 CBD:THC Treatment | 0.8165 | 0.4515 | 3.27 | *0.0705* |
| Alcohol x Stress | 0.5418 | 0.5222 | 1.08 | 0.2995 |
| Alcohol x Stress x CBD Treatment | -0.1980 | 0.7458 | 0.07 | 0.7907 |
| Alcohol x Stress x 3:1 CBD:THC Treatment | -0.4684 | 0.7108 | 0.43 | 0.5099 |
| ***Distance Traveled*** | | | | |
| Alcohol x CBD Treatment | 0.0012 | 0.1752 | 0.00 | 0.9947 |
| Alcohol x 3:1 CBD:THC Treatment | 0.0495 | 0.1754 | 0.08 | 0.7777 |
| Stress x CBD Treatment | 0.0698 | 0.1501 | 0.22 | 0.6419 |
| Stress x 3:1 CBD:THC Treatment | 0.2449 | 0.1378 | 3.16 | *0.0755* |
| Alcohol x Stress | 0.3115 | 0.1726 | 3.25 | *0.0712* |
| Alcohol x Stress x CBD Treatment | -0.3027 | 0.2495 | 1.47 | 0.2250 |
| Alcohol x Stress x 3:1 CBD:THC Treatment | -0.2760 | 0.2386 | 1.34 | 0.2473 |
| ***Time Immobile*** | | | | |
| Alcohol x CBD Treatment | 0.2403 | 0.3128 | 0.59 | 0.4424 |
| Alcohol x 3:1 CBD:THC Treatment | -0.1860 | 0.3028 | 0.38 | 0.5392 |
| Stress x CBD Treatment | 0.0264 | 0.2985 | 0.01 | 0.9294 |
| Stress x 3:1 CBD:THC Treatment | -0.4872 | 0.2724 | 3.20 | *0.0736* |
| Alcohol x Stress | -0.4049 | 0.3143 | 1.66 | 0.1976 |
| Alcohol x Stress x CBD Treatment | -0.0741 | 0.4418 | 0.03 | 0.8668 |
| Alcohol x Stress x 3:1 CBD:THC Treatment | 0.1597 | 0.4243 | 0.14 | 0.7067 |
| ***Ratio of Time Spent Immobile in Center*** | | | | |
| Alcohol x CBD Treatment | -29.6264 | 3.1321 | 198.59 | **<0.0001*** |
| Alcohol x 3:1 CBD:THC Treatment | -1.0786 | 2.9206 | 0.16 | 0.7119 |
| Stress x CBD Treatment | -1.2850 | 2.8074 | 0.18 | 0.6472 |
| Stress x 3:1 CBD:THC Treatment | -1.1015 | 2.4691 | 0.19 | 0.6555 |
| Alcohol x Stress | -0.2563 | 2.8412 | 0.01 | 0.9281 |
| Alcohol x Stress x CBD Treatment | 30.6294 | 0 | N/A | N/A |
| Alcohol x Stress x 3:1 CBD:THC Treatment | -0.4592 | 4.0053 | 0.03 | 0.9087 |
| ***S100β Counts*** | | | | |
| Alcohol x CBD Treatment | 0.2654 | 0.0907 |  | **0.0034*** |
| Alcohol x 3:1 CBD:THC Treatment | 0.2279 | 0.0941 |  | **0.0154*** |
| Stress x CBD Treatment | -0.0280 | 0.0780 |  | 0.7197 |
| Stress x 3:1 CBD:THC Treatment | 0.0231 | 0.0725 |  | 0.7505 |
| Alcohol x Stress | 0.0589 | 0.0985 |  | 0.5499 |
| Alcohol x Stress x CBD Treatment | -0.1138 | 0.1280 |  | 0.3741 |
| Alcohol x Stress x 3:1 CBD:THC Treatment | -0.1880 | 0.1312 |  | 0.1519 |
| ***Microglia Density*** | | | | |
| Alcohol x CBD Treatment | 0.0400 | 0.1112 |  | 0.7193 |
| Alcohol x 3:1 CBD:THC Treatment | -0.2215 | 0.1162 |  | *0.0566* |
| Stress x CBD Treatment | -0.0082 | 0.0996 |  | 0.9343 |
| Stress x 3:1 CBD:THC Treatment | -0.0314 | 0.0938 |  | 0.7377 |
| Alcohol x Stress | -0.1685 | 0.1204 |  | 0.1616 |
| Alcohol x Stress x CBD Treatment | 0.0509 | 0.1582 |  | 0.7476 |
| Alcohol x Stress x 3:1 CBD:THC Treatment | 0.2958 | 0.1621 |  | *0.0681* |
| ***Percent of Active Microglia*** | | | | |
| Alcohol x CBD Treatment | -0.3135 | 0.2661 |  | 0.2388 |
| Alcohol x 3:1 CBD:THC Treatment | -0.1408 | 0.2759 |  | 0.6098 |
| Stress x CBD Treatment | -0.0858 | 0.2392 |  | 0.7198 |
| Stress x 3:1 CBD:THC Treatment | -0.1774 | 0.2268 |  | 0.4339 |
| Alcohol x Stress | -0.3373 | 0.2892 |  | 0.2435 |
| Alcohol x Stress x CBD Treatment | 0.2154 | 0.3803 |  | 0.5711 |
| Alcohol x Stress x 3:1 CBD:THC Treatment | 0.1664 | 0.3891 |  | 0.6689 |

Supplemental Table 2 – Interaction effects of each of the outcomes in 24-hour withdrawal. The estimate values provide the effect size of the interactions. Bolded p-values with an asterisk show significant p-values, italicized p-values show trends of significance, although they do not approach significance.

| **24-hour Withdrawal Interaction Effects** | | | | |
| --- | --- | --- | --- | --- |
| **Parameter** | **Estimate** | **Standard Error** | **Wald Chi-Square** | **P-Value** |
| ***Time in Center*** | | | | |
| Alcohol x CBD Treatment | -0.3544 | 0.4068 |  | 0.3835 |
| Alcohol x 3:1 CBD:THC Treatment | 0.8084 | 0.3784 |  | **0.0326*** |
| Stress x CBD Treatment | 0.2372 | 0.4199 |  | 0.5722 |
| Stress x 3:1 CBD:THC Treatment | 0.8097 | 0.3859 |  | **0.0359*** |
| Alcohol x Stress | 0.1127 | 0.4107 |  | 0.7838 |
| Alcohol x Stress x CBD Treatment | 0.1879 | 0.5839 |  | 0.7475 |
| Alcohol x Stress x 3:1 CBD:THC Treatment | -0.9775 | 0.5364 |  | *0.0684* |
| ***Distance Traveled*** | | | | |
| Alcohol x CBD Treatment | 0.0103 | 0.1429 | 0.01 | 0.9427 |
| Alcohol x 3:1 CBD:THC Treatment | -0.1315 | 0.1351 | 0.95 | 0.3302 |
| Stress x CBD Treatment | 0.0693 | 0.1487 | 0.22 | 0.6410 |
| Stress x 3:1 CBD:THC Treatment | 0.2433 | 0.1364 | 3.18 | *0.0745* |
| Alcohol x Stress | 0.0402 | 0.1470 | 0.07 | 0.7845 |
| Alcohol x Stress x CBD Treatment | 0.0622 | 0.2080 | 0.09 | 0.7649 |
| Alcohol x Stress x 3:1 CBD:THC Treatment | -0.0133 | 0.1940 | 0.00 | 0.9454 |
| ***Time Immobile*** | | | | |
| Alcohol x CBD Treatment | 0.4073 | 0.3267 |  | 0.2125 |
| Alcohol x 3:1 CBD:THC Treatment | 0.0058 | 0.3019 |  | 0.9848 |
| Stress x CBD Treatment | 0.0166 | 0.3263 |  | 0.9595 |
| Stress x 3:1 CBD:THC Treatment | -0.4864 | 0.2980 |  | 0.1026 |
| Alcohol x Stress | 0.1026 | 0.3242 |  | 0.7516 |
| Alcohol x Stress x CBD Treatment | -0.8174 | 0.4608 |  | *0.0761* |
| Alcohol x Stress x 3:1 CBD:THC Treatment | -0.0862 | 0.4224 |  | 0.8383 |
| ***Ratio of Time Spent Immobile in Center*** | | | | |
| Alcohol x CBD Treatment | -0.3534 | 1.6305 | 0.07 | 0.7951 |
| Alcohol x 3:1 CBD:THC Treatment | -0.0629 | 1.2402 | 0.00 | 0.9596 |
| Stress x CBD Treatment | -0.6674 | 1.5729 | 0.18 | 0.6713 |
| Stress x 3:1 CBD:THC Treatment | -0.6635 | 1.4060 | 0.22 | 0.6370 |
| Alcohol x Stress | -2.5247 | 1.6575 | 2.32 | 0.1277 |
| Alcohol x Stress x CBD Treatment | 1.8004 | 2.1539 | 0.70 | 0.4032 |
| Alcohol x Stress x 3:1 CBD:THC Treatment | 2.0739 | 1.9333 | 1.15 | 0.2834 |
| ***S100β Counts*** | | | | |
| Alcohol x CBD Treatment | 0.1001 | 0.0978 |  | 0.3061 |
| Alcohol x 3:1 CBD:THC Treatment | 0.2384 | 0.0941 |  | **0.0113*** |
| Stress x CBD Treatment | -0.0416 | 0.0915 |  | 0.6495 |
| Stress x 3:1 CBD:THC Treatment | 0.0731 | 0.0926 |  | 0.4297 |
| Alcohol x Stress | 0.1108 | 0.1036 |  | 0.2849 |
| Alcohol x Stress x CBD Treatment | -0.1004 | 0.1395 |  | 0.4715 |
| Alcohol x Stress x 3:1 CBD:THC Treatment | -0.2714 | 0.1331 |  | **0.0414*** |
| ***Microglia Density*** | | | | |
| Alcohol x CBD Treatment | 0.0683 | 0.1129 |  | 0.5454 |
| Alcohol x 3:1 CBD:THC Treatment | 0.0396 | 0.1094 |  | 0.7175 |
| Stress x CBD Treatment | -0.0070 | 0.1043 |  | 0.9462 |
| Stress x 3:1 CBD:THC Treatment | -0.0310 | 0.0982 |  | 0.7524 |
| Alcohol x Stress | 0.0052 | 0.1255 |  | 0.9669 |
| Alcohol x Stress x CBD Treatment | -0.0228 | 0.1680 |  | 0.8920 |
| Alcohol x Stress x 3:1 CBD:THC Treatment | 0.0183 | 0.1557 |  | 0.9063 |
| ***Percent of Active Microglia*** | | | | |
| Alcohol x CBD Treatment | 0.2337 | 0.2488 |  | 0.3475 |
| Alcohol x 3:1 CBD:THC Treatment | 0.1320 | 0.2430 |  | 0.5868 |
| Stress x CBD Treatment | -0.0846 | 0.2284 |  | 0.7109 |
| Stress x 3:1 CBD:THC Treatment | -0.1768 | 0.2167 |  | 0.4146 |
| Alcohol x Stress | 0.4732 | 0.2745 |  | 0.0847 |
| Alcohol x Stress x CBD Treatment | -0.5960 | 0.3654 |  | 0.1028 |
| Alcohol x Stress x 3:1 CBD:THC Treatment | -0.3945 | 0.3410 |  | 0.2473 |

Supplemental Table 3 – Statistical evidence through the Bayesian Information Criterion (BIC) showing that negative binomial distribution model is as good as or better than normal or Poisson distribution in most cases in the outcomes measured for the 4-hour withdrawal.

| **BIC for 4-hour withdrawal** | |
| --- | --- |
| **Distribution** | **BIC** |
| ***Time in Center*** | |
| Normal | 1123.1563 |
| Poisson | 1766.4230 |
| Negative Binomial | 1065.4108 |
| ***Distance Traveled*** | |
| Normal | 908.4208 |
| Poisson | 945.2540 |
| Negative Binomial | 933.4947 |
| ***Time Immobile*** | |
| Normal | 1578.3095 |
| Poisson | 4334.8743 |
| Negative Binomial | 1522.1458 |
| ***Ratio of Time Spent Immobile in Center*** | |
| Normal | 577.0704 |
| Poisson | 355.7616 |
| Negative Binomial | 241.5858 |
| ***S100β Counts*** | |
| Normal | 976.0504 |
| Poisson | 979.6877 |
| Negative Binomial | 977.5417 |
| ***Microglia Density*** | |
| Normal | 1362.5806 |
| Poisson | 1945.8978 |
| Negative Binomial | 1365.8733 |
| ***Percent of Active Microglia*** | |
| Normal | 970.0415 |
| Poisson | 1155.9492 |
| Negative Binomial | 971.0872 |

Supplemental Table 4 – Statistical evidence through the Bayesian Information Criterion (BIC) showing that negative binomial distribution model is as good as or better than normal or Poisson distribution in most cases in the outcomes measured for the 24-hour withdrawal.

| **BIC for 24-hour withdrawal** | |
| --- | --- |
| **Distribution** | **BIC** |
| ***Time in Center*** | |
| Normal | 1434.4385 |
| Poisson | 2411.1027 |
| Negative Binomial | 1359.9065 |
| ***Distance Traveled*** | |
| Normal | 1023.1294 |
| Poisson | 1035.3217 |
| Negative Binomial | 1036.2157 |
| ***Time Immobile*** | |
| Normal | 1800.2317 |
| Poisson | 5034.1393 |
| Negative Binomial | 1702.8773 |
| ***Ratio of Time Spent Immobile in Center*** | |
| Normal | 984.7550 |
| Poisson | 686.4605 |
| Negative Binomial | 415.1970 |
| ***S100β Counts*** | |
| Normal | 946.4515 |
| Poisson | 955.5700 |
| Negative Binomial | 945.4115 |
| ***Microglia Density*** | |
| Normal | 1480.0975 |
| Poisson | 2168.8260 |
| Negative Binomial | 1487.6576 |
| ***Percent of Active Microglia*** | |
| Normal | 1051.0452 |
| Poisson | 1245.4786 |
| Negative Binomial | 1051.5044 |

Supplemental Table 5 – The least squares mean, standard error of the mean (SEM), and standard deviation (SD) values along with the arithmetic mean, SEM, and SD values for each condition and measure presented.

| **Alcohol Exposure** | **Stress Exposure** | **Treatment** | **Least Squares**  **Mean, SEM, SD** | **Arithmetic**  **Mean, SEM, SD** |
| --- | --- | --- | --- | --- |
| ***Time in Center*** | | | | |
|  |  |  |  |  |
| Air | No Stress | Vehicle | 18.18, 3.79, 13.13 | 18.37, 4.90, 16.98 |
|  |  | CBD | 17.59, 3.54, 12.76 | 17.38, 3.40, 12.27 |
|  |  | 3:1 CBD:THC | 12.66, 2.27, 9.36 | 12.71, 2.43, 10.02 |
|  | Stress | Vehicle | 10.39, 2.25, 7.79 | 10.45, 1.83, 6.34 |
|  |  | CBD | 12.74, 2.72, 9.44 | 12.56, 2.85, 9.87 |
|  |  | 3:1 CBD:THC | 16.26, 2.65, 11.83 | 16.18, 2.62, 11.71 |
| 4-hr withdrawal | No Stress | Vehicle | 13.00, 3.54, 11.19 | 13.40, 3.58, 11.32 |
|  |  | CBD | 6.41, 1.85, 5.84 | 6.28, 1.57, 4.95 |
|  |  | 3:1 CBD:THC | 8.65, 2.42, 7.64 | 9.22, 3.21, 10.16 |
|  | Stress | Vehicle | 12.80, 3.50, 11.08 | 12.49, 1.98, 6.27 |
|  |  | CBD | 6.57, 1.88, 5.94 | 6.73, 1.90, 5.99 |
|  |  | 3:1 CBD:THC | 12.07, 3.31, 10.47 | 11.81, 3.36, 10.64 |
| 24-hr withdrawal | No Stress | Vehicle | 29.88, 5.87, 21.14 | 30.42, 3.28, 11.81 |
|  |  | CBD | 20.29, 4.22, 14.63 | 19.90, 4.03, 13.96 |
|  |  | 3:1 CBD:THC | 46.72, 7.96, 32.80 | 45.73, 7.86, 32.39 |
|  | Stress | Vehicle | 19.11, 3.82, 13.77 | 19.38, 2.37, 8.54 |
|  |  | CBD | 19.85, 4.13, 14.32 | 19.54, 2.25, 7.81 |
|  |  | 3:1 CBD:THC | 25.27, 4.51, 18.05 | 24.77, 3.75, 14.99 |
| ***Distance Traveled*** | | | | |
|  |  |  |  |  |
| Air | No Stress | Vehicle | 19.18, 1.39, 4.81 | 19.01, 2.10, 7.26 |
|  |  | CBD | 18.86, 1.33, 4.78 | 18.47, 1.14, 4.10 |
|  |  | 3:1 CBD:THC | 18.20, 1.13, 4.67 | 17.97, 1.76, 7.27 |
|  | Stress | Vehicle | 16.00, 1.25, 4.33 | 15.80, 1.14, 3.96 |
|  |  | CBD | 16.87, 1.29, 4.47 | 16.67, 1.63, 5.64 |
|  |  | 3:1 CBD:THC | 19.37, 1.13, 5.06 | 17.60, 1.70, 7.58 |
| 4-hr withdrawal | No Stress | Vehicle | 12.58, 1.27, 4.01 | 12.17, 0.68, 2.15 |
|  |  | CBD | 12.38, 1.25, 3.94 | 12.21, 0.85, 2.68 |
|  |  | 3:1 CBD:THC | 12.54, 1.33, 4.22 | 12.01, 2.01, 6.36 |
|  | Stress | Vehicle | 14.33, 1.29, 4.07 | 14.40, 0.80, 2.54 |
|  |  | CBD | 11.17, 1.19, 3.75 | 10.95, 0.71, 2.23 |
|  |  | 3:1 CBD:THC | 13.85, 1.26, 4.00 | 13.91, 1.14, 3.61 |
| 24-hr withdrawal | No Stress | Vehicle | 18.64, 1.30, 4.68 | 18.94, 0.73, 2.65 |
|  |  | CBD | 18.52, 1.36, 4.72 | 18.26, 1.74, 6.04 |
|  |  | 3:1 CBD:THC | 15.51, 1.02, 4.22 | 15.71, 1.17, 4.83 |
|  | Stress | Vehicle | 16.19, 1.20, 4.31 | 16.45, 1.06, 3.81 |
|  |  | CBD | 18.35, 1.35, 4.69 | 18.08, 0.79, 2.72 |
|  |  | 3:1 CBD:THC | 16.95, 1.12, 4.47 | 16.82, 1.38, 5.52 |
| ***Time Immobile*** | | | | |
|  |  |  |  |  |
| Air | No Stress | Vehicle | 38.92, 6.51, 22.53 | 43.23, 9.49, 32.87 |
|  |  | CBD | 40.16, 6.42, 23.14 | 42.27, 6.14, 22.15 |
|  |  | 3:1 CBD:THC | 84.27, 11.59, 47.78 | 91.76, 16.73, 68.96 |
|  | Stress | Vehicle | 71.31, 11.68, 40.46 | 76.68, 11.40, 39.49 |
|  |  | CBD | 74.82, 12.24, 42.37 | 79.96, 11.77, 40.76 |
|  |  | 3:1 CBD:THC | 94.93, 11.97, 53.51 | 99.30, 17.85, 79.82 |
| 4-hr withdrawal | No Stress | Vehicle | 72.32, 11.85, 37.45 | 71.97, 6.99, 22.09 |
|  |  | CBD | 93.85, 15.35, 48.54 | 89.69, 9.84, 31.10 |
|  |  | 3:1 CBD:THC | 129.42, 20.95, 66.24 | 128.90, 21.68, 68.55 |
|  | Stress | Vehicle | 87.77, 14.31, 45.26 | 86.92, 5.32, 16.82 |
|  |  | CBD | 108.60, 17.62, 55.74 | 108.80, 11.49, 36.34 |
|  |  | 3:1 CBD:THC | 113.20, 18.38, 58.10 | 111.50, 11.63, 36.77 |
| 24-hr withdrawal | No Stress | Vehicle | 33.40, 5.39, 19.42 | 33.17, 3.38, 12.18 |
|  |  | CBD | 51.79, 8.54, 29.59 | 54.04, 12.72, 44.07 |
|  |  | 3:1 CBD:THC | 72.73, 10.00, 41.21 | 72.90, 11.10, 45.76 |
|  | Stress | Vehicle | 67.80, 10.67, 38.47 | 68.30, 8.15, 29.38 |
|  |  | CBD | 47.21, 7.82, 27.10 | 46.51, 5.31, 18.40 |
|  |  | 3:1 CBD:THC | 83.29, 11.76, 47.04 | 86.32, 13.26, 53.04 |
| ***Ratio of Time Spent Immobile in Center*** | | | | |
|  |  |  |  |  |
| Air | No Stress | Vehicle | 0.01, 0.01, 0.04 | 0.01, 0.01, 0.02 |
|  |  | CBD | 0.02, 0.02, 0.06 | 0.02, 0.02, 0.06 |
|  |  | 3:1 CBD:THC | 0.06, 0.03, 0.13 | 0.07, 0.06, 0.24 |
|  | Stress | Vehicle | 0.02, 0.02, 0.06 | 0.01, 0.01, 0.04 |
|  |  | CBD | 0.02, 0.01, 0.05 | 0.02, 0.02, 0.06 |
|  |  | 3:1 CBD:THC | 0.04, 0.02, 0.10 | 0.04, 0.02, 0.09 |
| 4-hr withdrawal | No Stress | Vehicle | 0.04, 0.03, 0.10 | 0.04, 0.03, 0.10 |
|  |  | CBD | 0.00, 0.00, 0.00 | 0.00, 0.00, 0.00 |
|  |  | 3:1 CBD:THC | 0.07, 0.07, 0.20 | 0.06, 0.04, 0.11 |
|  | Stress | Vehicle | 0.05, 0.04, 0.14 | 0.04, 0.03, 0.09 |
|  |  | CBD | 0.10, 0.09, 0.28 | 0.05, 0.05, 0.16 |
|  |  | 3:1 CBD:THC | 0.03, 0.03, 0.08 | 0.03, 0.03, 0.09 |
| 24-hr withdrawal | No Stress | Vehicle | 0.05, 0.03, 0.11 | 0.03, 0.02, 0.07 |
|  |  | CBD | 0.06, 0.04, 0.13 | 0.05, 0.02, 0.08 |
|  |  | 3:1 CBD:THC | 0.18, 0.08, 0.34 | 0.19, 0.05, 0.20 |
|  | Stress | Vehicle | 0.01, 0.01, 0.02 | 0.01, 0.00, 0.01 |
|  |  | CBD | 0.02, 0.01, 0.05 | 0.03, 0.02, 0.08 |
|  |  | 3:1 CBD:THC | 0.08, 0.04, 0.17 | 0.10, 0.05, 0.19 |
| ***S100ß Counts*** | | | | |
|  |  |  |  |  |
| Air | No Stress | Vehicle | 71.01, 3.42, 11.34 | 70.99, 2.16, 7.15 |
|  |  | CBD | 70.07, 2.94, 10.60 | 70.09, 2.56, 9.23 |
|  |  | 3:1 CBD:THC | 65.60, 2.82, 11.26 | 69.02, 2.59, 10.36 |
|  | Stress | Vehicle | 70.83, 3.41, 11.32 | 69.85, 3.23, 10.70 |
|  |  | CBD | 67.05, 2.98, 9.88 | 69.85, 3.23, 10.70 |
|  |  | 3:1 CBD:THC | 70.40, 3.23, 14.08 | 69.62, 1.81, 7.90 |
| 4-hr withdrawal | No Stress | Vehicle | 55.91, 3.33, 8.16 | 55.94, 4.19, 10.27 |
|  |  | CBD | 72.28, 3.00, 9.49 | 72.29, 3.35, 10.60 |
|  |  | 3:1 CBD:THC | 68.36, 3.48, 9.20 | 68.08, 4.55, 12.03 |
|  | Stress | Vehicle | 58.32, 3.17, 8.38 | 58.11, 4.29, 11.36 |
|  |  | CBD | 65.43, 2.99, 8.96 | 65.24, 2.76, 8.28 |
|  |  | 3:1 CBD:THC | 60.47, 3.24, 8.56 | 60.21, 4.64, 12.28 |
| 24-hr withdrawal | No Stress | Vehicle | 65.32, 3.67, 9.72 | 65.14, 6.78, 17.93 |
|  |  | CBD | 71.24, 3.48, 10.43 | 70.69, 3.39, 10.18 |
|  |  | 3:1 CBD:THC | 76.59, 3.03, 10.91 | 76.15, 4.01, 14.44 |
|  | Stress | Vehicle | 72.78, 3.94, 10.43 | 72.62, 3.19, 8.43 |
|  |  | CBD | 68.88, 3.59, 10.15 | 68.41, 4.37, 12.36 |
|  |  | 3:1 CBD:THC | 69.99, 2.75, 10.30 | 69.56, 1.92, 7.19 |
| ***Microglia Density*** | | | | |
|  |  |  |  |  |
| Air | No Stress | Vehicle | 265.33, 14.20, 47.10 | 266.70, 12.73, 42.21 |
|  |  | CBD | 259.27, 12.40, 46.40 | 263.00, 15.91, 59.52 |
|  |  | 3:1 CBD:THC | 255.10, 11.36, 45.41 | 256.50, 12.42, 49.67 |
|  | Stress | Vehicle | 270.80, 15.18, 47.99 | 271.30, 16.70, 52.80 |
|  |  | CBD | 262.75, 13.48, 46.70 | 264.50, 15.18, 52.58 |
|  |  | 3:1 CBD:THC | 252.41, 10.31, 44.94 | 252.60, 9.02, 39.30 |
| 4-hr withdrawal | No Stress | Vehicle | 258.72, 17.93, 43.91 | 258.60, 16.98, 41.59 |
|  |  | CBD | 263.77, 14.14, 44.72 | 263.40, 10.44, 33.01 |
|  |  | 3:1 CBD:THC | 199.35, 13.04, 34.49 | 199.80, 16.59, 43.88 |
|  | Stress | Vehicle | 222.89, 14.46, 38.24 | 224.00, 10.31, 27.27 |
|  |  | CBD | 237.16, 13.50, 40.50 | 238.00, 11.54, 34.61 |
|  |  | 3:1 CBD:THC | 223.71, 14.50, 38.36 | 224.10, 11.59, 30.66 |
| 24-hr withdrawal | No Stress | Vehicle | 261.89, 17.59, 46.54 | 263.60, 17.23, 45.59 |
|  |  | CBD | 273.98, 15.51, 49.03 | 279.50, 15.94, 50.40 |
|  |  | 3:1 CBD:THC | 261.96, 13.49, 46.73 | 265.20, 13.44, 46.57 |
|  | Stress | Vehicle | 268.68, 19.49, 47.74 | 272.10, 7.02, 17.20 |
|  |  | CBD | 272.82, 18.32, 48.47 | 276.60, 19.85, 52.51 |
|  |  | 3:1 CBD:THC | 265.37, 12.71, 47.54 | 269.80, 13.83, 51.73 |
| ***Percent of Active Microglia*** | | | | |
|  |  |  |  |  |
| Air | No Stress | Vehicle | 21.90, 2.60, 8.64 | 21.91, 2.22, 7.37 |
|  |  | CBD | 26.45, 2.74, 10.24 | 26.54, 2.21, 8.27 |
|  |  | 3:1 CBD:THC | 24.01, 2.34, 9.35 | 24.03, 2.23, 8.92 |
|  | Stress | Vehicle | 23.88, 2.94, 9.31 | 23.90, 3.41, 10.77 |
|  |  | CBD | 26.50, 2.95, 10.21 | 26.57, 2.85, 9.87 |
|  |  | 3:1 CBD:THC | 21.94, 1.98, 8.65 | 21.95, 2.08, 9.06 |
| 4-hr withdrawal | No Stress | Vehicle | 26.96, 4.42, 10.84 | 26.96, 3.79, 9.29 |
|  |  | CBD | 23.87, 3.08, 9.74 | 23.87, 3.28, 10.38 |
|  |  | 3:1 CBD:THC | 25.69, 3.93, 10.39 | 25.69, 3.93, 10.41 |
|  | Stress | Vehicle | 20.99, 3.29, 8.71 | 20.99, 3.67, 9.70 |
|  |  | CBD | 21.15, 2.93, 8.79 | 21.15, 3.01, 9.04 |
|  |  | 3:1 CBD:THC | 19.77, 3.13, 8.28 | 19.77, 5.78, 15.30 |
| 24-hr withdrawal | No Stress | Vehicle | 19.85, 3.00, 7.95 | 19.87, 2.38, 6.29 |
|  |  | CBD | 30.29, 3.67, 11.62 | 30.47, 4.45, 14.07 |
|  |  | 3:1 CBD:THC | 24.84, 2.78, 9.65 | 24.90, 3.02, 10.46 |
|  | Stress | Vehicle | 34.75, 5.29, 12.96 | 34.83, 5.20, 12.75 |
|  |  | CBD | 26.85, 3.91, 10.34 | 26.95, 4.49, 11.88 |
|  |  | 3:1 CBD:THC | 24.56, 2.58, 9.65 | 24.67, 2.18, 8.15 |
